# Peripheral γδ T-cell remodeling in psoriasis is associated with effector-biased transcriptional organization and reduced representation of a circulating *CSMD1*-marked state

**DOI:** 10.3389/fimmu.2026.1885325

**Published:** 2026-08-20

**Authors:** Nanxi Shi, Yiming Chen, Jiale Tang, Sihan Chen, Xi Lin, Yingying Ye, Jingsong Zhang, Ruili Chen, Jiaqi Zeng, Guodong Sun, Xiaoqiao Yang, Suhua Zhong, Fang Huang, Zhenhua Li, Xin Tang, Shi Wu, Yunting Liang

**Affiliations:** 1 The Biomedical Translational Research Institute, Key Laboratory of Ministry of Education for Viral Pathogenesis & Infection Prevention and Control, School of Life Science and Technology, Jinan University, Guangzhou, China; 2 The Biomedical Translational Research Institute, Key Laboratory of Ministry of Education for Viral Pathogenesis & Infection Prevention and Control, School of Medicine, Jinan University, Guangzhou, China; 3 School of Life Science and Biopharmaceutics, Guangdong Pharmaceutical University, Guangzhou, China; 4 Department of Systems Biomedical Sciences, School of Medicine, Jinan University, Guangzhou, China; 5 Institute of Ecological Sciences, School of Life Sciences, South China Normal University, Guangzhou, China; 6 Zhuhai People’s Hospital (The Affiliated Hospital of Beijing Institute of Technology, Zhuhai Clinical Medical College of Jinan University), Jinan University, Zhuhai, China; 7 Guangdong Provincial Key Laboratory of Spine and Spinal Cord Reconstruction, The Fifth Affiliated Hospital of Jinan University, Heyuan, China; 8 Department of Gastroenterology, The Affiliated Guangdong Second Provincial General Hospital of Jinan University, Guangzhou, China; 9 Department of Computer Science, Guangdong Institute of Smart Education, Jinan University, Guangzhou, China; 10 Department of Dermatology, The First Affiliated Hospital of Jinan University, Guangzhou, China

**Keywords:** cell heterogeneity, peripheral blood, psoriasis, scRNA-seq, γδ T cell

## Abstract

**Background:**

Despite the prominent immune dysregulation in psoriasis, the organization of peripheral blood γδ T cell states remains incompletely defined.

**Methods:**

Here, we performed single-cell transcriptomic profiling of enriched peripheral blood-derived γδ T cells from healthy donors and patients with psoriasis. By integrating state annotation, inferred transcriptional ordering, ligand–receptor communication, transcriptome–inferred metabolite–sensor communication, and regulatory network analyses, we constructed a circulating γδ T-cell state atlas.

**Results:**

We identified eight γδ T-cell states. Within the resolution of this dataset, psoriasis-derived cells did not form a clearly separated disease-exclusive state; instead, disease-associated changes were mainly reflected by altered representation and transcriptional organization of pre-existing states, including reduced peripheral representation of a *CSMD1*-marked γδ T-cell state. Pseudotime analysis arranged γδ T cells along an inferred activation- and cytotoxicity-associated transcriptional continuum, with Naive-like cells at the lower-pseudotime region and cytotoxic effector-, NK-like-, cytotoxic memory-like-, terminal-branch-, and *CSMD1*-marked states toward higher-pseudotime regions. Psoriasis-derived γδ T cells were more frequent in cytotoxicity-related regions. This pattern suggests a shift toward effector states. In parallel, peripheral γδ T cell communication suggested cytotoxic-state-biased ligand–receptor patterns, with rewiring of MIF-related and metabolic signaling and reduced network participation of *CSMD1*^+^ γδ T cells. Regulatory analyses further linked FOXP1, STAT1, and NFATC3 as candidate regulators associated with early-like, transitional/*CSMD1*-marked, and cytotoxic-associated transcriptional states, respectively.

**Conclusions:**

These findings support a state-resolved framework of circulating γδ T-cell remodeling in psoriasis and identify reduction and possible redistribution of a *CSMD1*-marked late-stage state as a feature of disease-associated immune remodeling.

## 1 Introduction

Psoriasis is a chronic inflammatory skin disease in which dysregulated immune cell–keratinocyte interactions converge on the IL-23/IL-17 axis, a central pathogenic pathway and a validated therapeutic target (1–3). Although single-cell and spatial studies have substantially advanced our understanding of lesional immune circuits in psoriatic skin, these efforts have focused mainly on αβ T cells and local tissue niches (4–6). By contrast, how circulating immune states are organized and remodeled in psoriasis remains less well understood.

γδ T cells are innate-like lymphocytes with rapid effector potential and marked functional heterogeneity across tissues and differentiation states. Beyond conventional classification by TCR usage, they span multiple cell states, including naïve-like, transitional, activated, and cytotoxic programs (7, 8). They also participate in diverse signaling networks involving CLEC2D–CD161, HLA-E–CD94/NKG2, and MIF-related receptor axes (9–12). In psoriasis, γδ T cells have been implicated as important contributors to IL-17 production, inflammatory memory, and metabolic adaptation, particularly in lesional settings (7, 13–15). However, the cellular landscape of peripheral blood-derived γδ T cells, their differentiation relationships, and their communication architecture in psoriasis remain poorly defined (16).

Recent advances in single-cell and epigenomic systems analysis now make it possible to address these questions by integrating transcriptomic state mapping with trajectory inference, ligand–receptor communication analysis, and regulon reconstruction (17–20). These approaches additionally provide a framework to examine whether metabolically associated signaling is selectively rewired across γδ T-cell states in disease (21–23). A key unresolved issue is therefore whether psoriasis generates new peripheral γδ T-cell populations or instead reshapes the distribution and functional wiring of pre-existing states, particularly along transitional stages linking early and terminal programs.

Thus, we performed single-cell transcriptomic profiling of FACS-purified peripheral blood γδ T cells from psoriasis patients and healthy donors. We identified eight circulating γδ T-cell states and found that psoriasis was associated with altered representation of pre-existing states, accompanied by lower representation of a *CSMD1*-marked transcriptional state. Integrated analyses of pseudotime, cell–cell communication, metabolic signaling, and transcriptional regulation further supported an effector-skewed organization of circulating γδ T cells and nominated candidate communication and regulatory programs associated with disease-related remodeling. Together, these findings provide a state-resolved framework for circulating γδ T-cell remodeling in psoriasis. They also nominate reduced peripheral representation of a *CSMD1*-marked state as a candidate feature that requires validation in larger cohorts.

## 2 Methods

### 2.1 Human subjects and samples

Peripheral blood was collected from patients with clinician-diagnosed moderate-to-severe plaque psoriasis recruited from the Department of Dermatology, Zhuhai People’s Hospital, and from age- and sex-matched healthy controls. For consistency with figure labels, healthy control donors are referred to as the Normal group throughout the manuscript. The study was approved by the Ethics Committee of Zhuhai People’s Hospital (LYXM2025-H-084), and written informed consent was obtained from all participants. Peripheral blood samples were collected in EDTA anticoagulant tubes (lavender-top), and PBMCs were isolated within 2 h of collection for single-cell transcriptomic analysis. Samples from the normal group (n = 3) and patients with psoriasis (n = 3) were processed for downstream γδ T-cell enrichment and sequencing. Donor-level clinical information for the PBMC cohort is summarized in **Supplementary Table 1**, including age, sex, diagnosis, disease duration, Psoriasis Area and Severity Index (PASI), body surface area (BSA), Dermatology Life Quality Index (DLQI), treatment status, recent therapies, comorbidities, infection status, and sample date. All patients had plaque psoriasis/psoriasis vulgaris without documented autoimmune or major systemic diseases. None had received biologic or systemic therapy prior to sampling; prior treatment was limited to topical corticosteroids and/or calcineurin inhibitors. Exclusion criteria included major internal disease, infection, neuropsychiatric disorders, malignancy, immunodeficiency, pregnancy or lactation, prior systemic therapy, or phototherapy/topical treatment within 4 weeks before sampling.

For tissue validation, FFPE skin specimens were retrieved from the pathology archive of Zhuhai People’s Hospital and sectioned specifically for multiplex immunofluorescence (mIF). One tissue block was analyzed per donor, including Normal skin (n = 3) and psoriatic lesional skin (n = 3). For each donor, the entire tissue section was fully imaged under identical acquisition settings, and the quantified tissue area was used to generate one donor-level value after exclusion of tissue folds, detached tissue regions, surface crusts, hair follicles, and poorly preserved areas.

### 2.2 PBMC isolation and γδ T-cell sorting

Peripheral blood mononuclear cells (PBMCs) were isolated via density-gradient centrifugation and stained at 4 °C with anti-human CD45-PE-Cy7 (clone HI30, BioLegend, #304016), anti-human CD3-FITC (clone UCHT1, BioLegend, #300406), and anti-human TCRγδ-BV421 (clone B1, BioLegend, #331224), along with 7-AAD (MultiSciences, Hangzhou, China) for live/dead discrimination. After exclusion of doublets and 7-AAD^+^ dead cells, live CD45^+^ lymphocytes were gated, and CD3^+^ TCRγδ^+^ cells were sorted by flow cytometry to ≥ 95% purity, with post-sort viability ≥ 85%.

### 2.3 Single-cell library preparation and sequencing

Single cells were encapsulated, and 3′ mRNA libraries were prepared on the MobiNova-100 droplet system using MobiCube 3′ kits, following the manufacturer’ s protocol. Libraries were sequenced on Illumina platforms, targeting ~6–10 k cells per sample and ≥ 40–60 k read pairs per cell.

### 2.4 Preprocessing, integration, and clustering

Raw single-cell transcriptomic count matrices were imported into Seurat (v4.4.0) for downstream analysis. Cells expressing fewer than 200 or more than 6,000 genes, with fewer than 1,000 or more than 80,000 UMIs, or with >15% mitochondrial transcripts were excluded. Cells with high hemoglobin transcript expression were also removed to minimize contamination from residual erythrocytes. Doublet detection was performed independently for each sample before data integration. For each sample, cells were normalized, highly variable genes were identified, data were scaled, and principal component analysis (PCA) was performed. Low-resolution graph-based clustering was then conducted at resolution 0.3 to support sample-specific doublet prediction. The expected number of doublets was determined according to the number of recovered cells for each sample, and doublets were predicted using DoubletFinder. Only predicted singlets were retained for downstream integration, clustering, cell-state annotation, and subsequent analyses. Donor-level quality-control metrics, DoubletFinder-derived doublet counts, doublet-removal rates, and retained cell numbers are summarized in **Supplementary Table 2**.

### 2.5 Cell–cell communication

Classical ligand–receptor-based cell–cell communication among γδ T-cell states in Normal and Psoriasis samples was inferred using CellChat v2.1.2. Group-specific CellChat objects were generated from annotated γδ T-cell subsets, and communication networks were constructed based on state-level ligand and receptor expression profiles. Inferred communication patterns were summarized by sender, receiver, and global interaction properties across γδ T-cell states. Differences between groups were descriptively compared in terms of interaction number, information flow, and pathway composition to assess psoriasis-associated changes in transcriptome-inferred communication patterns. For the main CellChat analysis, no downsampling or cell-number normalization was performed before network inference. CellChat was applied to condition-level pooled γδ T-cell objects after quality control and cell-state annotation, preserving the original cell numbers of each annotated γδ T-cell state. As a sensitivity analysis, CellChat was repeated after cell-state-matched downsampling, in which each annotated γδ T-cell state was downsampled to 719 cells per group before rerunning CellChat.

### 2.6 Analysis of external PBMC and skin single-cell datasets

External single-cell datasets were analyzed to provide exploratory context for γδ T-cell and *CSMD1* transcript detection in peripheral blood and psoriatic skin. For GSE194315, the processed expression matrix and accompanying cell-type annotations provided by the original study were used, as this dataset had already undergone quality control, dimensionality reduction, clustering, and cell-type annotation. Major immune cell populations were retained according to the original metadata, and *CSMD1* expression was assessed within the annotated γδ T-cell compartment, without establishing a new *CSMD1*^+^ γδ T-cell population in the public dataset. For GSE151177, raw count matrices were reprocessed following the workflow described in the corresponding publication. Quality control, normalization, dimensionality reduction, MNN-based batch correction, clustering, and UMAP visualization were performed using parameters matched to the original study whenever applicable, including the number of principal components, batch-correction strategy, and clustering resolution. Cell-type annotation was performed according to marker genes reported in the original publication and verified using canonical lineage markers. Minor differences in cluster boundaries were observed, likely due to software-version differences and updates in MNN implementation; however, the major cell-type identities and biological interpretation were broadly concordant with the original study. The processed annotations were then used for downstream CellChat analysis centered on *CSMD1*^+^ γδ T cells.

### 2.7 Metabolism-associated communication analysis (mCCC)

Metabolite-mediated cell–cell communication (mCCC) was inferred using MEBOCOST v1.0.4. The annotated γδ T-cell states were used as cell groups through the celltype field, and the organism was set to human. MEBOCOST was run using the built-in mebocost.conf configuration file and the corresponding enzyme–metabolite–sensor relationship database distributed with the package. This database links metabolic enzymes, inferred metabolites, and metabolite sensors based on curated public resources. Metabolite abundance was estimated from the expression of metabolic enzymes using the mebocost method, and all sensor categories were included.

For filtering, automatic cutoffs were applied to both sensor expression and inferred metabolite abundance (cutoff_exp = “auto” and cutoff_met = “auto”), excluding molecules ranked in the lowest 25% across cells. A minimum detection proportion of 15% was required for the relevant sensor or inferred metabolite signal in at least one cell group (cutoff_prop = 0.15). Statistical significance of inferred metabolite–sensor communication events was assessed using permutation testing with 1,000 random shuffles (n_shuffle = 1000, random seed = 12345). Sender or receiver cell groups containing fewer than 10 cells were excluded from the analysis. Multiple testing was controlled using the Benjamini–Hochberg method, and interactions with permutation_test_fdr ≤ 0.05 were retained.

To further constrain the inferred directionality of metabolite-associated interactions, COMPASS was used to estimate cell-state-level metabolic flux tendencies from transcriptomic data. Automatic efflux and influx cutoffs (efflux_cut = “auto” and influx_cut = “auto”) were applied, and only metabolite–sensor interactions with COMPASS-inferred secretion and uptake directions consistent with the predicted flux tendencies were retained. The final mCCC network used for downstream analysis and visualization therefore included interactions passing both the permutation-test FDR threshold and the COMPASS-inferred flux-consistency filter. Because MEBOCOST and COMPASS analyses were based on transcriptomic inference, these results were interpreted as candidate metabolite-associated communication signatures rather than direct measurements of metabolite abundance, metabolite transfer, metabolic flux, or functional metabolic regulation.

### 2.8 Trajectory and developmental potency

To characterize the Monocle2-inferred activation- and cytotoxicity-associated transcriptional continuum, pseudotime analysis was performed using Monocle2 (v2.24.0). Ordering genes were selected from differentially expressed genes and state-associated markers. The root was assigned based on the early/naive-like features of the Naive-like state. Pseudotime distributions were used to compare the relative positioning of annotated γδ T-cell states and donor groups. CytoTRACE (v0.3.3) was used as a complementary analysis of predicted cellular ordering, not as independent evidence of lineage direction (24).

### 2.9 Gene regulatory network inference and regulon-level readouts

To resolve the transcriptional regulatory programs associated with γδ T cell state transitions, gene regulatory network inference was performed within a SCENIC/regulon-based framework to examine regulatory programs associated with annotated γδ T-cell states. Regulon activity was assessed at the single-cell and state levels to define state specificity and changes along the inferred transcriptional continuum. Regulon specificity score (RSS), regulon co-activity relationships, and module analyses were used to identify candidate transcription factors associated with early-like, transitional activation-associated, and cytotoxic effector-associated states.

### 2.10 In silico transcription factor perturbation analysis

CellOracle v0.20.0 was used to simulate FOXP1, STAT1, and NFATC3 perturbations. The base GRN was generated from the CellOracle human promoter GRN, using JASPAR 2020 and HOCOMOCO v11 motifs. Promoters were defined as −500 to +100 bp around the transcription start site. No enhancer, scATAC-seq, or chromatin-accessibility data were used. State-specific GRNs were inferred by ridge regression with α = 10. TF-target links were retained at F-test P < 0.05 and R² > 0.01. Knockout was simulated by setting TF expression to 0. Overexpression was simulated within the observed expression range. Perturbation effects were propagated with n_propagation = 3 and interpreted as inferred regulatory associations, not causal validation.

### 2.11 Multiplex immunofluorescence

Formalin-fixed, paraffin-embedded (FFPE) skin sections (5–7 μm) were used for multiplex immunofluorescence (mIF). The mIF skin samples were not paired with the PBMC samples used for single-cell RNA-seq. Sections were deparaffinized, rehydrated, and subjected to heat-induced epitope retrieval using citrate buffer (pH 6.0) for CSMD1 and TCRδ, and Tris–EDTA buffer (pH 9.0) for GZMK. After blocking, sections were stained by sequential primary antibody incubation followed by HRP-mediated tyramide signal amplification (TSA). Primary antibodies included rabbit anti-CSMD1 (PA5-50645, Invitrogen/Thermo Fisher Scientific), mouse anti-GZMK (clone 1C3A4, 67272-1-Ig, Proteintech), and rabbit anti-TCRδ/TRDC (clone EPR27043-16, ab313573, Abcam). Antibodies were titrated within the manufacturer-recommended range for FFPE staining, typically 1:100–1:400.

For sequential TSA staining, each staining round was followed by HRP quenching and heat-mediated antibody stripping before the next primary antibody incubation. These steps were used to reduce antibody carryover between staining rounds, particularly because CSMD1 and TCRδ antibodies were both rabbit-derived. Additional antibody-control slides were not available for this archived mIF set; this limitation was acknowledged, and mIF results were interpreted cautiously.

For each donor, the entire tissue section was fully imaged at 20× magnification under identical exposure settings. Quantification was performed on the full imaged tissue section rather than on selected microscopic fields. Tissue folds, detached tissue regions, surface crusts, hair follicles, and poorly preserved areas were excluded from the quantified tissue area. Representative high-magnification merged and single-channel images were generated from selected regions to visualize cellular co-detection of TCRδ, CSMD1, and GZMK.

Image quantification was performed in QuPath. DAPI-based nuclear segmentation followed by cell expansion was used to define individual cell objects. Cell-level fluorescence intensities for TCRδ, CSMD1, and GZMK were extracted, and marker-positive cells were defined using a uniform threshold of >10 arbitrary fluorescence units across all images. Because additional antibody-control slides were unavailable, this threshold-based analysis was used as an object-level co-detection assessment rather than definitive proof of antigen co-expression. TCRδ^+^CSMD1^+^GZMK^+^ cells and total TCRδ^+^ cells were quantified as cells per mm² using the quantified tissue area of the full imaged section. The percentage of TCRδ^+^CSMD1^+^GZMK^+^ cells among total TCRδ^+^ cells was also calculated. For each donor, the fully imaged tissue section generated one donor-level value, and group comparisons were performed at the donor level.

### 2.12 Statistics, software, and reproducibility

Single-cell data processing and visualization were performed primarily in R (v4.3.3), using Seurat (v4.4.0), CellChat (v2.1.2), Monocle2 (v2.24.0), CytoTRACE (v0.3.3). mCCC analysis and TF perturbation analysis were performed in Python using mebocost (v1.2.2) and CellOracle (v0.20.0), respectively. Unless otherwise specified, analyses were performed using default parameters implemented in each package. Appropriate multiple-testing correction methods were applied where relevant, and statistical significance was defined as *P* < 0.05 or false discovery rate (FDR) < 0.05. *P*-value coding in figures: *P* < 0.05 (*), *P* < 0.01 (**), *P* < 0.001 (***).

## 3 Results

### 3.1 Reduced representation of a *CSMD1*-marked γδ T-cell state in peripheral blood from patients with psoriasis

**Figure 1:**
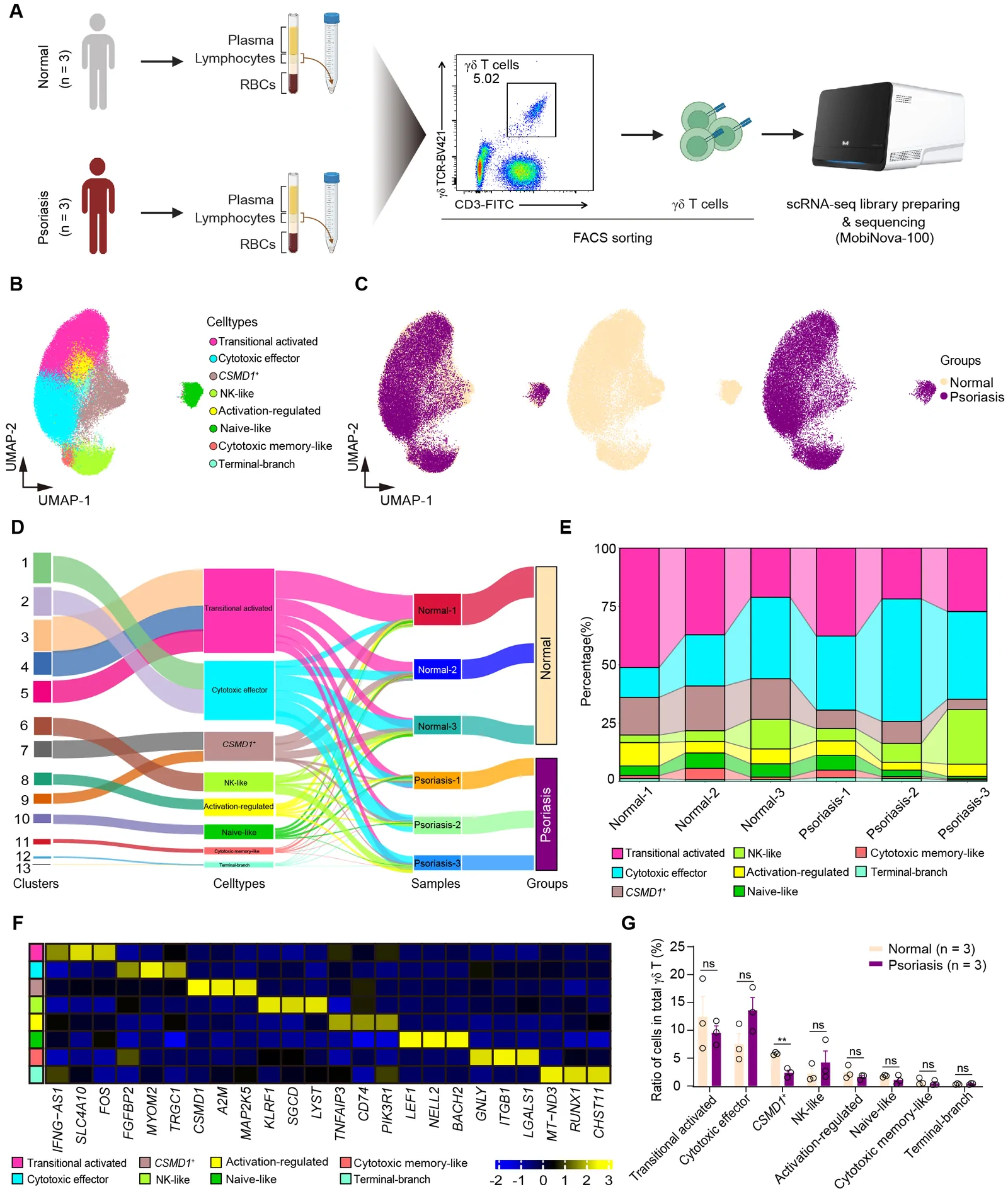
scRNA-seq reveals altered cell-state composition of circulating peripheral blood γδ T cells in psoriasis. **(A)** Schematic overview of the study design. PBMCs were collected from healthy donors (Normal, n = 3) and patients with psoriasis (Psoriasis, n = 3). CD3^+^TCRγδ^+^ γδ T cells were enriched by FACS and subjected to scRNA-seq using the MobiNova-100 platform. **(B)** UMAP visualization of integrated γδ T cells colored by cell-state annotation. Eight circulating γδ T-cell states were identified based on differentially expressed genes and representative transcriptional programs: Transitional activated, Cytotoxic effector, *CSMD1*^+^, NK-like, Activation-regulated, Naive-like, Cytotoxic memory-like, and Terminal-branch γδ T cells. **(C)** UMAP colored by group identity (Normal versus Psoriasis), showing the distribution of cells from the two groups in the integrated embedding space. **(D)** Sankey plot linking unsupervised clusters, annotated γδ T-cell states, individual donors and group identity. **(E)** Cellular composition of the eight γδ T-cell states across individual donors. **(F)** Heat map showing top three representative marker genes supporting state annotation across the eight γδ T-cell. **(G)** Donor-level quantification of the proportion of each γδ T-cell state within total retained γδ T cells. Bars indicate group means and error bars indicate SEM. Each dot represents one donor (n = 3 per group). Cell-state abundance was analyzed at the donor level rather than the single-cell level. Exact P values and Benjamini–Hochberg-adjusted q values are provided in **Supplementary Table 3**. Donor-level comparisons were performed as descriptive statistical analyses, with multiple testing controlled across the eight γδ T-cell states using the Benjamini–Hochberg method. *P < 0.05, **P < 0.01, ns, no significance.

To define how peripheral γδ T cells are transcriptionally organized in psoriasis, we performed single-cell RNA sequencing of FACS-enriched γδ T cells from peripheral blood mononuclear cells of three healthy donors and three patients with psoriasis. Because γδ T cells represent a relatively small fraction of circulating lymphocytes, CD3^+^TCRγδ^+^ γδ T cells were first enriched by fluorescence-activated cell sorting and subsequently subjected to scRNA-seq using the MobiNova-100 platform (**Figure 1A**; **Supplementary Figure 1A**). Before sorting, γδ T cells represented a detectable fraction of circulating CD3^+^ T cells, and post-sort analysis confirmed efficient enrichment of a highly purified γδ T-cell population (**Supplementary Figure 1A**). Across all samples, key quality-control parameters, including the number of detected genes, total UMI counts, and mitochondrial transcript proportions, were comparable between donors and remained stable after filtering low-quality cells (**Supplementary Figures 1B–D**), supporting the robustness of the dataset for downstream analysis.

Unsupervised clustering initially resolved 13 transcriptional clusters among peripheral γδ T cells. To move beyond cluster identity and capture biologically interpretable cell states, we integrated shared marker expression patterns and inferred functional programs, consolidating these clusters into eight major γδ T-cell states. These included Naive-like cells marked by early or circulating-associated gene programs, Transitional activated and Activation-regulated cells enriched for activation- and cytokine-response-related signatures, Cytotoxic effector and Cytotoxic memory-like cells characterized by cytotoxic molecules, NK-like cells expressing NK receptor-associated features, a *CSMD1*^+^ state marked by restricted *CSMD1* expression; and a Terminal-branch state enriched for late effector-associated genes (**Figure 1F**). Thus, circulating γδ T cells were not transcriptionally uniform, but instead occupied a structured landscape spanning naive-like, activated, cytotoxic, NK-like, CSMD1-marked, and terminal-branch-associated states.

Additional lineage-marker assessment supported the assignment of these annotated states within the γδ T-cell compartment. The annotated states retained core T-cell and γδ TCR-associated marker expression, including CD3D, CD3E, CD3G, TRDC, TRGC1, and TRGC2. Exploratory TRDV/TRGV transcripts and αβ T/MAIT/NKT-like markers were also examined to assess TCR-usage patterns and potential contamination, but were not used for definitive Vδ/Vγ subset assignment (**Supplementary Figure 7**; **Supplementary Table 4**).

We next examined whether psoriasis-derived γδ T cells formed a clearly separated disease-associated structure in the integrated embedding. In the UMAP colored by disease group, γδ T cells from Normal and Psoriasis donors were broadly intermixed, with no clear disease-driven segregation at the current sampling depth (**Figure 1C**). Within the resolution of this dataset, psoriasis-derived cells did not form a clearly separated disease-exclusive cluster or annotated γδ T-cell state. Instead, cells from each donor contributed to multiple transcriptional states (**Figure 1D**), suggesting that the main differences captured here were more likely reflected by altered representation and transcriptional organization of annotated pre-existing γδ T-cell states.

Consistent with this possibility, donor-level compositional analysis showed lower representation of the *CSMD1*-marked γδ T-cell state in patients with psoriasis. Among the eight annotated states, the *CSMD1*-marked γδ T-cell state showed lower donor-level representation in psoriasis samples (**Figures 1E, G**; **Supplementary Table 3**). In contrast, the remaining major γδ T-cell states did not show statistically significant differences at the current sample size (**Figure 1G**). Within this small cohort, the *CSMD1*-marked state represented the most evident donor-level compositional difference.

An additional Milo neighborhood differential abundance analysis showed a concordant lower-abundance tendency in *CSMD1*-marked neighborhoods, supporting this observation as a donor-aware sensitivity result rather than a single-cell-level statistical effect (**Supplementary Figure 2**). Because *CSMD1* is not a canonical γδ T-cell marker and was sparsely detected, we further assessed the technical robustness of this state. *CSMD1*-associated signals overlapped with core T-cell and γδ TCR-associated markers, were not associated with elevated doublet scores or non-T-cell lineage markers, and remained spatially restricted under increasing *CSMD1* expression thresholds (**Supplementary Figures 3–S5**; **Supplementary Tables 5–S7**).

Together, these data establish an eight-state transcriptional atlas of human peripheral blood γδ T cells and suggest reduced representation of a *CSMD1*-marked state in psoriasis. We next asked where this state was positioned along the inferred γδ T-cell transcriptional continuum.

### 3.2 Circulating γδ T cells are biased toward terminal cytotoxic-associated states in psoriasis

**Figure 2:**
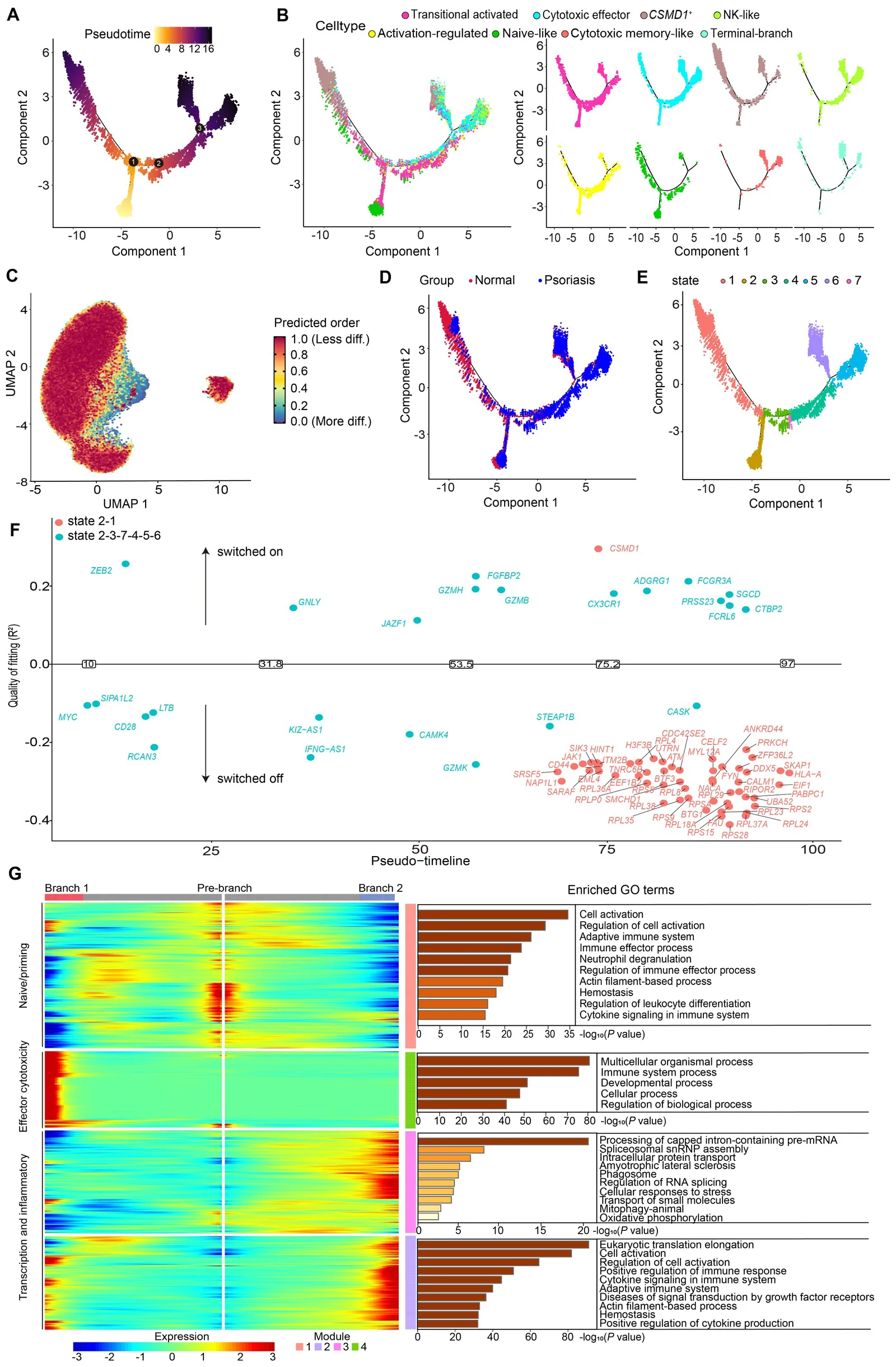
Circulating γδ T cells are arranged along a Monocle2-inferred activation- and cytotoxicity-associated transcriptional continuum in psoriasis. **(A)** Pseudotime ordering of circulating γδ T cells projected onto the trajectory space. Cells are colored by inferred pseudotime, with increasing color intensity indicating inferred ordering along the transcriptional continuum. **(B)** Annotated γδ T-cell states overlaid on the pseudotime trajectory. The integrated panel shows all eight states, and the separated panels show the distribution and preferential localization of individual states along the trajectory. **(C)** CytoTRACE-based prediction of cellular ordering across the trajectory. In this visualization, higher predicted-order values correspond to earlier, less differentiated states, whereas lower values correspond to later, more differentiated effector-associated states. **(D)** Distribution of γδ T cells from Normal and Psoriasis groups along the same trajectory. Cells are colored by disease group to visualize group-associated differences in pseudotime occupancy. **(E)** Partitioning of the trajectory into seven pseudotime states based on branch topology. State labels indicate trajectory-defined regions rather than annotated γδ T-cell subsets. **(F)** Distinct switching genes along the two trajectory branches were plotted onto the pseudo-timeline using GeneSwitches. The y axis indicates McFadden’s pseudo-R², and representative switched-on and switched-off genes are labeled. **(G)** Pseudotime expression modules were identified from dynamically expressed genes along the γδ T-cell state-transition continuum. GO terms of each module are shown on the right, with significance represented as −log_10_(*P* value).

To examine transcriptional relationships among circulating γδ T-cell states, we ordered individual γδ T cells along pseudotime. This analysis organized γδ T cells along a continuous trajectory that could be broadly divided into lower-pseudotime, activation-associated, and cytotoxicity-associated regions. Naive-like γδ T cells were positioned toward the lower-pseudotime region, followed by activation-associated regions enriched for Transitional activated and Activation-regulated γδ T cells. Higher-pseudotime regions contained *CSMD1*-marked, Cytotoxic memory-like, Cytotoxic effector, NK-like, and Terminal-branch γδ T-cell states (**Figures 2A, B**; **Supplementary Figure 8A**). CytoTRACE analysis showed a concordant gradient of predicted differentiation potential, with higher scores in lower-pseudotime regions and lower scores in cytotoxicity-associated regions (**Figure 2C**).

The assignment of the Naive-like state as the lower-pseudotime reference was supported by enrichment of early/naive-associated markers, including *TCF7*, *LEF1*, *BACH2*, *SELL*, *CCR7*, and *NELL2*, in the same region of the γδ T-cell manifold (**Supplementary Figure 9**).

At the population level, cells from the Normal group were more frequently distributed in lower-pseudotime regions, whereas psoriasis-derived cells were relatively enriched in higher-pseudotime, cytotoxicity-associated regions (**Figure 2D**). These findings suggest that circulating γδ T cells in psoriasis are associated with a relative transcriptional shift toward cytotoxic effector-associated programs. Trajectory partitioning further revealed a branch point at State 2, from which one minor branch terminated at State 1, whereas the major branch extended through States 3, 7, 4, and 5 before reaching State 6 (**Figure 2E**). When overlaid with cell-state annotations, this structure was consistent with a transcriptional gradient from Naive-like γδ T cells through activation-associated states and toward cytotoxic, NK-like, Terminal-branch, and *CSMD1*-marked programs.

Donor-resolved trajectory visualization further showed that this group-level tendency was not attributable to a single donor, although inter-donor variability was present (**Supplementary Figure 8B**). To evaluate whether the inferred ordering was driven primarily by *CSMD1* or a small set of major state-defining markers, Monocle2 trajectory inference was repeated after excluding *CSMD1* and the top representative marker genes from each annotated γδ T-cell state before ordering-gene selection. The major organization of the inferred transcriptional continuum was retained after this marker-exclusion procedure (**Supplementary Figure 10**).

Genes varying along pseudotime further supported this activation- and cytotoxicity-associated continuum (**Figures 2F, G**). Lower-pseudotime cells preferentially expressed genes associated with circulating T-cell programs, including *MYC*, *CD28*, and *LTB*. Activation-associated regions showed increased expression of genes such as *GZMK*, *IFNG*-*AS1*, and *CAMK4*, whereas higher-pseudotime regions were associated with ZEB2 and cytotoxic or migratory genes, including *GNLY*, *GZMB*, *GZMH*, *FGFBP2*, *CX3CR1*, and *FCGR3A*. GO enrichment of late dynamic modules further highlighted cytotoxic function, RNA processing, translation, and energy metabolism, consistent with cytotoxic effector-associated transcriptional programs. Notably, *CSMD1* displayed a late-induction pattern along pseudotime, remaining low in early cells and increasing only after activation-associated and cytotoxicity-associated programs had been initiated. Consistently, *CSMD1*-marked γδ T cells were positioned toward a higher-pseudotime segment rather than within the Naive-like or early activation-associated regions. These observations support interpreting the *CSMD1*-marked state as a restricted higher-pseudotime γδ T-cell transcriptional state. Thus, psoriasis-associated γδ T-cell remodeling involves enrichment of cytotoxicity-associated transcriptional programs, accompanied by reduced representation of the *CSMD1*-marked state in peripheral blood.

### 3.3 Transcriptome-inferred γδ T-cell communication changes and MIF-associated signals in psoriasis

To further characterize alterations in communication across γδ T cell states, we used CellChat to infer sender–receiver roles and their associated signaling pathways. Relative to the normal group, psoriasis samples exhibited a greater number of inferred interactions within the γδ T cell compartment, accompanied by a modest reduction in overall interaction strength (**Supplementary Figure 11A**). At the pathway level, γδ T cell subsets displayed clear sender–receiver specialization: several cytotoxic-associated subsets showed stronger outgoing activity across multiple pathways, whereas Naive-like and activation-associated states were relatively enriched on the receiving side (**Supplementary Figures 11B, D**). Network structures for the CLEC, MHC-I, MIF, and TGF-β pathways, together with contribution analyses of representative ligand–receptor pairs, including CLEC2D–KLRB1, are shown in **Supplementary Figures 11C–E**.

**Figure 3:**
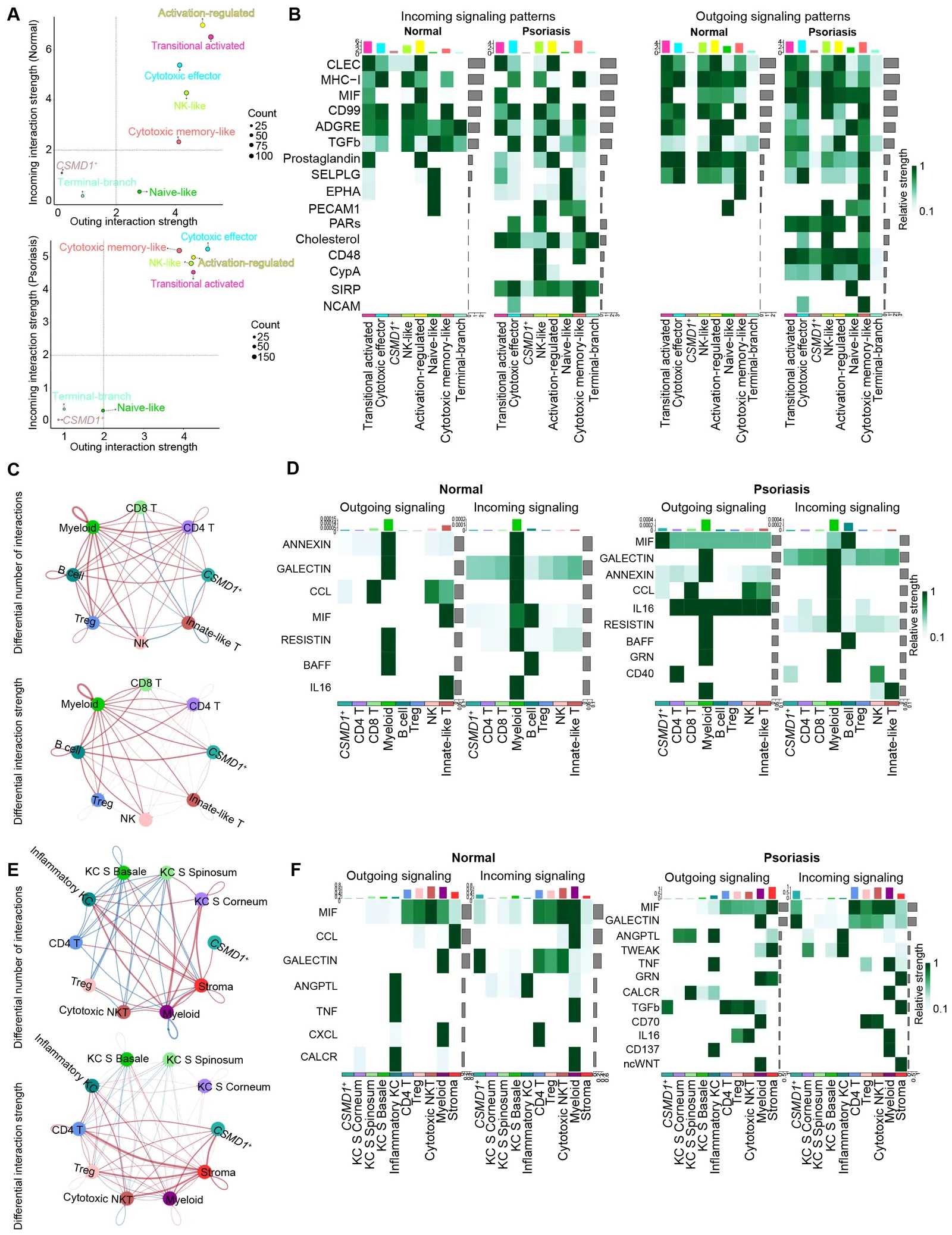
Transcriptome-inferred communication patterns involving *CSMD1*-expressing γδ T cells and mIF-based tissue context in psoriasis. **(A)** Global communication roles of γδ T-cell subsets inferred by CellChat. Normal (top) and psoriasis (bottom) groups are shown for comparison. **(B)** Heatmaps showing pathway-level incoming and outgoing communication patterns among γδ T-cell states in Normal and Psoriasis samples. Color intensity indicates relative signaling strength across γδ T-cell states. **(C)** Differential communication network centered on *CSMD1*^+^ γδ T cells in PBMCs, showing interactions with major peripheral immune cell types, including CD4 T cells, CD8 T cells, NK cells, B cells, myeloid cells, Treg cells and innate-like T cells. The upper panel shows differential interaction number and the lower panel shows differential interaction strength. Edge color indicates increased or decreased communication in psoriasis. **(D)** Heatmaps of representative signaling pathways mediating outgoing and incoming communication between *CSMD1*^+^ γδ T cells and other immune cell types in PBMCs. Normal and psoriasis groups are shown side by side. **(E)** Differential communication network centered on *CSMD1*^+^ γδ T cells in skin, showing interactions with major skin cell populations, including keratinocyte subsets, inflammatory cells, myeloid cells, stromal cells, CD4 T cells, Treg cells and cytotoxic NKT cells. The upper panel shows differential interaction number and the lower panel shows differential interaction strength. **(F)** Heatmaps of representative outgoing and incoming signaling pathways associated with *CSMD1*^+^ γδ T cells in skin from normal and psoriasis samples, showing differences in pathway composition across interacting skin cell types.

We first examined inferred communication roles among circulating γδ T-cell states in our discovery dataset (**Figures 3A, B**). In the γδ T-cell-restricted network, cytotoxic-associated states occupied more sender-biased positions in psoriasis, whereas Naive-like and activation-associated states remained relatively receiver-biased. By contrast, *CSMD1*-marked γδ T cells did not emerge as a dominant inferred sender or receiver within the intra-γδ T-cell network. Because the *CSMD1*-marked state showed limited inferred sender and receiver dominance within the γδ T-cell-restricted network, we next examined external PBMC and skin datasets as exploratory transcriptomic context. These public datasets were not γδ T cell-enriched, and *CSMD1* detection was sparse despite detectable *TRDC*-positive cells, limiting the robustness of *CSMD1*-centered analyses (**Supplementary Figure 12**; **Supplementary Table 9**). Using an external psoriasis peripheral blood reference dataset (GSE194315), we reconstructed transcriptome-inferred communication networks centered on *CSMD1*-expressing γδ T cells across major immune cell types. In psoriasis samples, these cells showed broader inferred connectivity with myeloid cells, CD4 T cells, CD8 T cells, B cells, NK cells, and innate-like T cells (**Figures 3C, D**). This network expansion was accompanied by enhanced MIF-related outgoing signaling and additional IL16-, GRN-, and CD40-related inferred modules, suggesting that *CSMD1*-expressing γδ T cells may be associated with a broader inflammatory communication context in peripheral blood.

Analysis of the public human skin single-cell dataset (GSE151177) revealed a related pattern in psoriatic skin (**Figures 3E, F**). *CSMD1*-expressing γδ T-cell-associated inferred interactions were observed with stromal populations, myeloid cells, and cytotoxic NKT cells, together with MIF-associated incoming signals and TGF-β-associated outgoing signals. Thus, although *CSMD1*-marked γδ T cells did not act as a dominant inferred communication hub within the γδ T-cell-restricted network, external datasets suggested that *CSMD1*-expressing γδ T cells may be embedded in broader inflammation- and tissue-remodeling-associated transcriptomic communication contexts in psoriasis.

Multiplex immunofluorescence provided supportive tissue-level context for TCRδ, GZMK, and CSMD1 staining patterns in normal skin and psoriatic lesions (**Supplementary Figure 13**). Psoriatic lesions showed higher relative fluorescence intensities of TCRδ, GZMK, and CSMD1 under identical imaging conditions. QuPath-based single-cell segmentation further identified TCRδ^+^CSMD1^+^GZMK^+^ cells in lesional tissue and enabled donor-level quantification of their tissue density. Although inter-donor variability was evident, TCRδ^+^CSMD1^+^GZMK^+^ cells and TCRδ^+^ cells were more readily detected in psoriatic lesions than in normal skin. Representative high-magnification images supported cellular co-detection of TCRδ, CSMD1, and GZMK signals. These findings support increased tissue-level TCRδ-, GZMK-, and CSMD1-associated signals in psoriatic lesions and are consistent with the presence of TCRδ^+^CSMD1^+^GZMK^+^ cells in lesional skin.

### 3.4 Transcriptome-inferred mCCC patterns are concentrated around activated and cytotoxic effector-associated γδ T-cell states in psoriasis

Under a conventional ligand–receptor (LR) framework, we observed changes in transcriptome-inferred communication patterns across γδ T-cell states. To explore whether metabolism-associated gene-expression programs showed related state-specific organization, we applied mCCC to infer candidate metabolite–sensor communication signatures from single-cell transcriptomic data.

**Figure 4:**
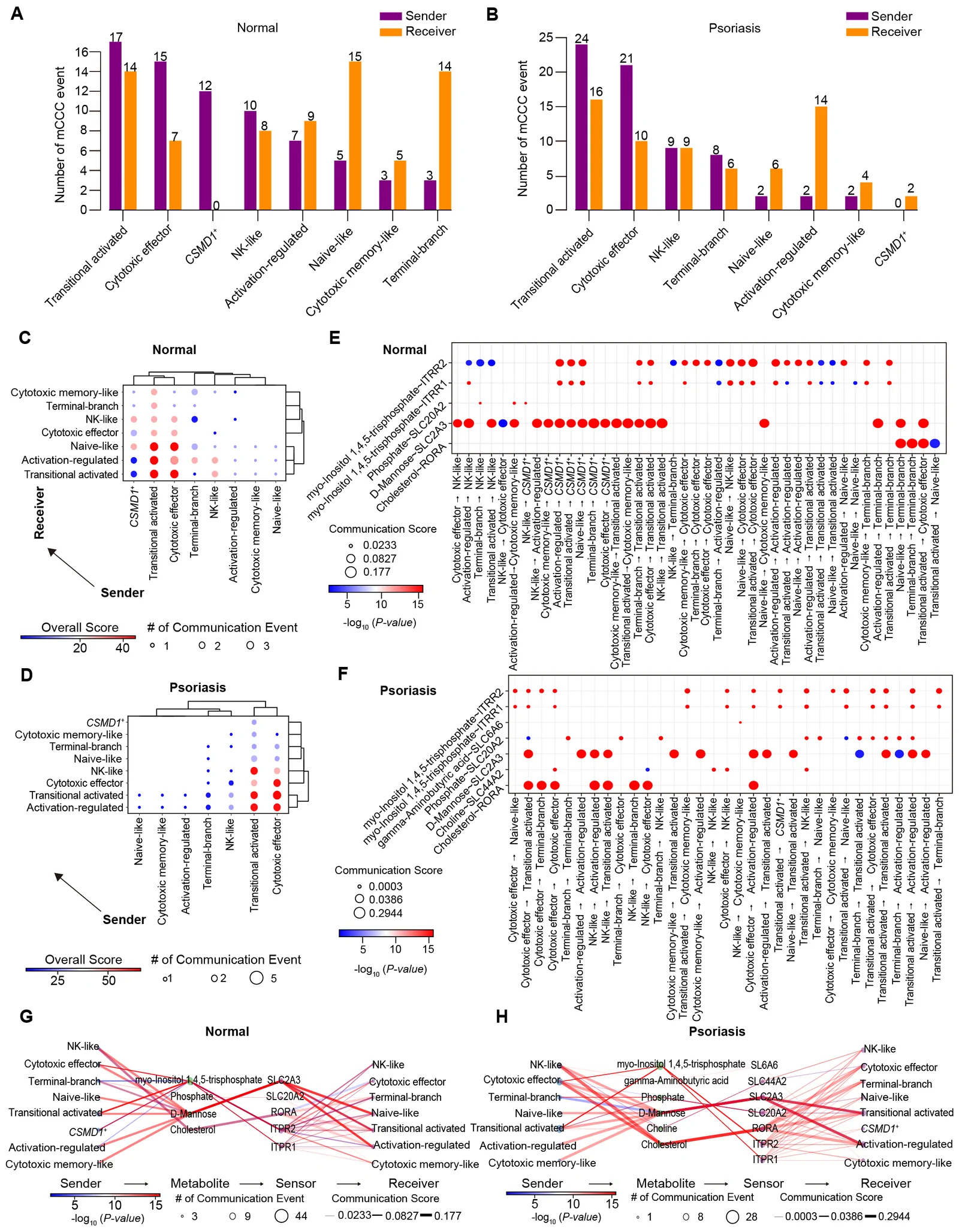
Comparison of metabolism-associated communication networks among circulating γδ T-cell states in normal and psoriasis samples. **(A, B)** Bar plots showing the number of significant metabolism-associated communication events detected for each γδ T-cell state as sender or receiver cells in Normal **(A)** and Psoriasis **(B)** samples. Purple indicates sender events, and orange indicates receiver events. The y axis indicates the number of inferred mCCC events for each state. **(C, D)** Dot plots showing the inferred mCCC events between each pair of γδ T-cell states in Normal **(C)** and Psoriasis **(D)** samples. Columns indicate sender states, and rows indicate receiver states. Dot size indicates the number of significant metabolite–sensor communications, and dot color represents the overall communication score for each sender–receiver pair. Hierarchical clustering highlights similarities in pairwise communication patterns. **(E, F)** Dot plots showing the distribution of representative significant metabolite–sensor/transporter axes across pairwise γδ T-cell state combinations in Normal **(E)** and Psoriasis **(F)** samples. Rows indicate representative metabolite-associated signaling axes, including myo-inositol 1,4,5-trisphosphate–ITPR1/2, γ-aminobutyrate–SLC6A6, D-mannose–SLC2A3, phosphate–SLC20A2, and cholesterol–RORA, and columns indicate sender-to-receiver state pairs. Dot size represents the communication score, and dot color represents statistical significance as −log_10_(*P* value). Only significant axes are displayed. **(G, H)** Network-style summaries of representative metabolism-associated communication routes in Normal **(G)** and Psoriasis **(H)** samples, linking sender states (left), metabolites, sensors/transporters, and receiver states (right). Edge color denotes statistical significance (−log_10_(*P* value)). Node size reflects the number of communication events, and the quantitative scale for the receiver side indicates the receiver communication score. Compared with the relatively dispersed network architecture observed in normal samples, psoriasis samples show a more concentrated metabolism-associated communication pattern, with stronger involvement of activated and terminal effector-related γδ T-cell states.

We first compared sender and receiver participation across the eight circulating γδ T cell states in mCCC events (**Figures 4A, B**). Overall, both groups showed state-specific sender–receiver biases rather than a uniform sender or receiver pattern. In Normal samples, sender events were most frequent in Transitional activated, Cytotoxic effector, *CSMD1*^+^, and NK-like γδ T cells, whereas receiver events were prominent in Naive-like, Transitional activated, Terminal-branch, and Activation-regulated γδ T cells. Notably, *CSMD1*^+^ γδ T cells showed a sender-biased pattern in Normal samples, with detectable sender but no receiver events. In Psoriasis samples, mCCC sender participation became more concentrated in Transitional activated and Cytotoxic effector γδ T cells. Receiver events were most frequent in Transitional activated and Activation-regulated γδ T cells, with additional involvement of Cytotoxic effector, NK-like, Terminal-branch, and Naive-like states. Compared with Normal samples, *CSMD1*^+^ γδ T cells showed markedly reduced mCCC participation and lost the sender-biased pattern observed in Normal samples. These results suggest a psoriasis-associated redistribution of transcriptome-inferred metabolite–sensor communication roles, with retained involvement of transitional and cytotoxic effector-associated states and reduced *CSMD1*-associated participation. We next examined inferred communication scores between each pair of γδ T-cell states (**Figures 4C, D**). In Normal samples, high-scoring mCCC events were relatively dispersed across multiple sender–receiver combinations. In Psoriasis samples, the strongest inferred scores were concentrated in interactions from Transitional activated γδ T cells to terminal effector-related receiver states, most prominently Cytotoxic effector and NK-like γδ T cells. By comparison, Naive-like γδ T cells showed limited involvement in high-scoring mCCC events, and *CSMD1*^+^ γδ T cells did not emerge as a major high-score communication hub. These observations suggest that the psoriasis-associated mCCC pattern involved not only differences in event counts across individual γδ T-cell states, but also a redistribution of inferred scores across specific sender–receiver pairs.

To further define the candidate molecular axes underlying this pattern, we analyzed significant metabolite–sensor/transporter axes across γδ T-cell state pairs (**Figures 4E, F**). Compared with Normal samples, Psoriasis samples showed a more restricted distribution of significant metabolite–sensor communications, with enrichment in sender–receiver pairs involving Transitional activated γδ T cells and terminal effector-related states. Several axes emerged from this analysis, including D-mannose–SLC2A3, cholesterol–RORA, phosphate–SLC20A2, and IP_3_–ITPR1/2. Additional choline- and GABA-related axes were also detected in Psoriasis samples, together with sensors or transporters such as SLC44A2 and SLC6A6. Representative metabolite–sensor communication routes for selected sender–receiver pairs and their group-wise differences are shown in **Supplementary Figure 14**. Network-style summaries further illustrated the inferred relationships among sender states, metabolites, sensors or transporters, and receiver states in Normal and Psoriasis samples (**Figures 4G, H**). In Normal samples, metabolite–sensor communications were distributed across a broader set of γδ T-cell state combinations. In Psoriasis samples, the network became more focused around activated-to-terminal effector communication routes, with Transitional activated and Activation-regulated γδ T cells acting as prominent sender states and terminal effector-related γδ T cells acting as major receiver states. Among the detected metabolite–sensor axes, D-mannose–SLC2A3, cholesterol–RORA, and IP_3_–ITPR1/2 showed stronger contributions to the psoriasis-associated network.

Altogether, these results suggest that psoriasis is associated with a shift in transcriptome-inferred mCCC patterns from a relatively dispersed organization toward a more concentrated architecture centered on activated-to-cytotoxic effector-associated γδ T-cell interactions. This shift involved changes in inferred sender–receiver participation, state-pair communication scores, and the distribution of candidate metabolite–sensor or metabolite–transporter axes.

### 3.5 FOXP1, STAT1, and NFATC3 associated regulatory patterns along the inferred γδ T cell trajectory

**Figure 5:**
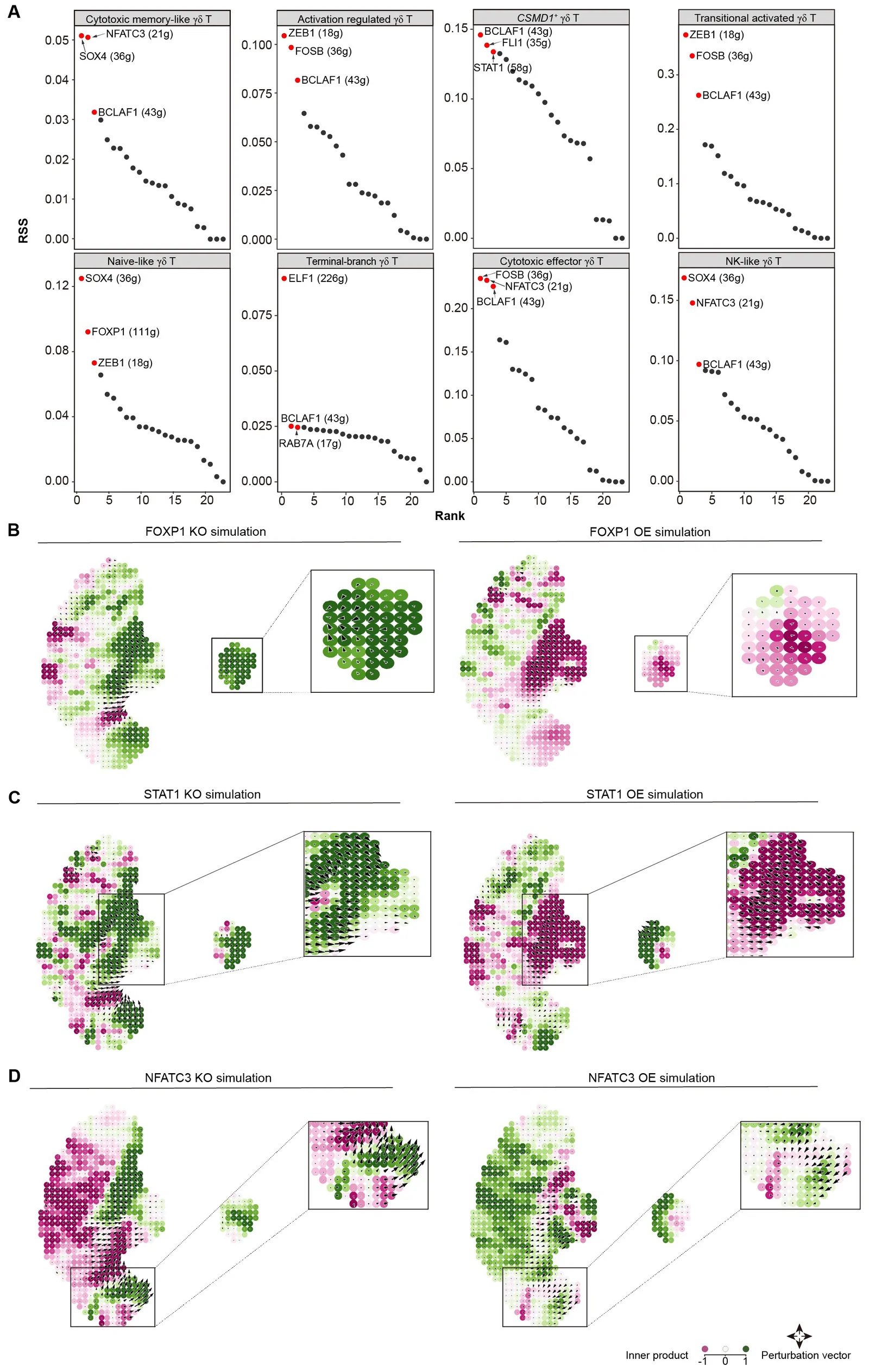
Candidate transcription factor modules and in silico perturbation-associated vector patterns along the inferred γδ T-cell continuum. **(A)** Regulon specificity ranking (RSS) across γδ T-cell states. Each panel corresponds to one subset, including Cytotoxic memory-like, Activation-regulated, *CSMD1*^+^, Transitional activated, Naive-like, Terminal-branch, Cytotoxic effector and NK-like γδ T cells. The x axis indicates regulon rank and the y axis indicates RSS value. Selected regulons with high state specificity are labeled; numbers in parentheses indicate target gene counts. **(B–D)** Vector-field and in silico perturbation analysis in the pseudotime/state-transition space, showing the predicted directional effects of key transcription factors on cell-state movement. Each row shows knockout (KO) and overexpression (OE) simulations for FOXP1 **(B)**, STAT1 **(C)** and NFATC3 **(D)**. Points or hexagons indicate cell positions in low-dimensional space, and black arrows indicate perturbation vectors. Insets show local vector changes in selected regions. The color scale represents the inner product between perturbation vectors and the reference pseudotime/state axis, indicating the degree of directional alignment.

To identify transcriptional regulators associated with different regions of the inferred γδ T cell trajectory, we examined regulon co-activity patterns and their state-specific distribution across γδ T cell subsets (**Figure 5A**; **Supplementary Figures 15–17**). Connection specificity index (CSI) analysis revealed modular reorganization of the co-active regulon modules that varied across the inferred state continuum, and RSS analysis identified transcription factors most representative of individual states. Integrating these features with pseudotime positioning placed Naive-like γδ T cells at the lower pseudotime values, *CSMD1^+^* γδ T cells toward a late-stage *CSMD1*-marked region, and Terminal-branch γδ T, Cytotoxic effector γδ T, NK-like γδ T, and Cytotoxic memory-like γδ T cells at the higher-pseudotime and branch-associated regions. On this basis, FOXP1, STAT1, and NFATC3 were selected as candidate regulators associated with early, transitional, and terminal effector-associated transcriptional states, respectively.

CellOracle-based in silico perturbation simulations revealed region-restricted effects that closely matched the state specificity of the corresponding regulons (**Figures 5B–D**). FOXP1 perturbation was confined primarily to the early trajectory region occupied by Naive-like γδ T cells. Simulated FOXP1 loss produced vectors aligned with the local pseudotime gradient, consistent with inferred shift away from the early-state program, whereas FOXP1 overexpression produced the opposite pattern, consistent with an association between FOXP1 activity and preservation of an early-like transcriptional state. These findings support FOXP1 as a candidate regulator associated with early-like state maintenance consistent with prior evidence that FOXP1 maintains naïve T cell quiescence and restrains effector differentiation (25, 26). STAT1 perturbation was enriched across the activation-to-late transition region, spanning activation-associated states and the higher-pseudotime *CSMD1*-marked region segment of the trajectory. STAT1 loss produced vectors aligned with the local pseudotime gradient, consistent with continued progression toward downstream states. By contrast, STAT1 overexpression generated vectors opposing the local pseudotime gradient and pointing back toward the connecting region, suggesting maintenance of, or bias toward, a transitional state rather than progression toward the branch terminus. This interpretation is broadly consistent with previous evidence linking STAT1 to interferon-responsive T-cell activation and differentiation programs (27), although the present analysis does not establish a causal role for STAT1 in γδ T-cell state transitions. In contrast, simulated NFATC3 perturbation was localized mainly to higher-pseudotime regions and terminal effector-associated branches. Simulated NFATC3 loss and overexpression produced opposing vector patterns within these regions. Together with the high NFATC3 regulon specificity observed in several terminal and cytotoxic-associated subsets, these findings identify NFATC3 as a candidate regulator associated with terminal effector-related transcriptional programs, consistent with established roles of NFAT signaling in T-cell activation and effector differentiation (28).

Collectively, the regulon and in silico perturbation analyses suggest state-associated regulatory patterns along the inferred γδ T-cell trajectory. FOXP1 activity was preferentially associated with early-like states, STAT1 with intermediate activation-associated regions, and NFATC3 with terminal effector-associated states.

### 3.6 Integrated model of peripheral γδ T-cell remodeling in psoriasis

**Figure 6:**
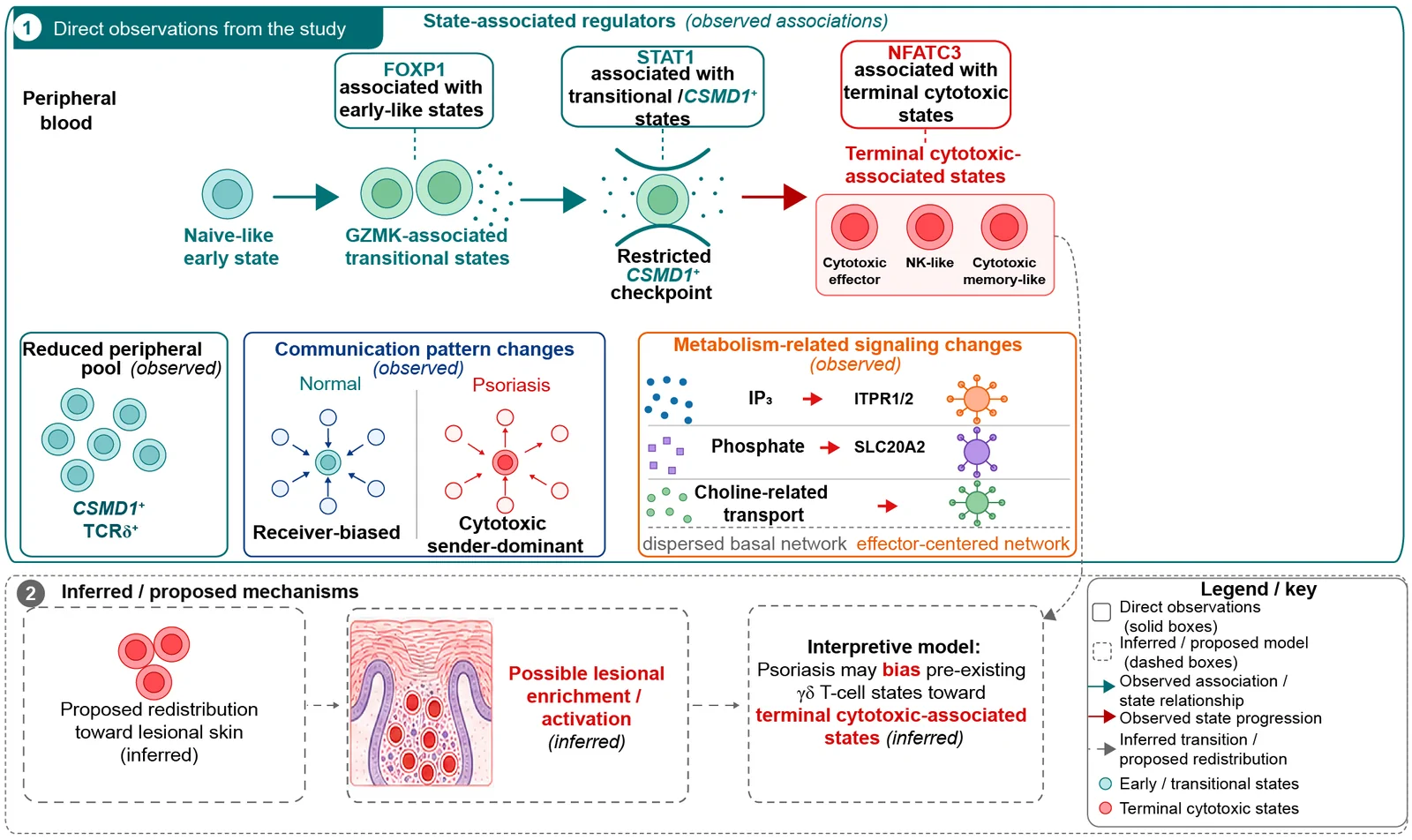
Integrated model of peripheral γδ T-cell remodeling in psoriasis. Schematic summary separating study-derived observations/associations from inferred or proposed mechanisms. Psoriasis was associated with remodeling of pre-existing peripheral γδ T-cell states, including reduced donor-level abundance of a restricted *CSMD1*-marked state and a shift toward cytotoxic effector-associated programs. Regulon-based analyses nominated FOXP1, STAT1, and NFATC3 as candidate regulators of early-like, transitional/*CSMD*1-marked, and terminal cytotoxic-associated states. Transcriptome-inferred communication and metabolism-associated analyses suggested cytotoxic sender-dominant and effector-centered network changes. Stronger *CSMD1*-associated signals in psoriatic skin are compatible with blood–skin compartmental differences, but lesional enrichment or redistribution remains inferential and requires direct validation. Solid boxes indicate study-derived observations or associations; dashed boxes indicate inferred or proposed mechanisms.

By integrating cell-state composition, inferred pseudotime structure, communication patterns, metabolic signatures, and regulon associations, we constructed a schematic model of peripheral γδ T-cell remodeling in psoriasis (**Figure 6**), distinguishing observed findings from inferred mechanisms.

In peripheral blood, γδ T cells in psoriasis did not form a distinct disease-specific population but showed altered representation of pre-existing states. Naive-like cells occupied lower-pseudotime regions, whereas cytotoxic effector, NK-like, memory-like, and terminal states were enriched at higher pseudotime. The *CSMD1*-marked state localized to a restricted higher-pseudotime region and showed reduced abundance in psoriasis.

These changes were accompanied by coordinated shifts in inferred functional programs. CellChat suggested a transition from receiver-biased interactions in controls to cytotoxic sender-dominant patterns in psoriasis. Metabolism-associated analysis indicated that candidate signatures (e.g., IP_3_–ITPR1/2, phosphate–SLC20A2, choline-related axes) became more concentrated in effector states. Regulon and perturbation analyses nominated FOXP1, STAT1, and NFATC3 as candidate regulators of early-like, transitional, *CSMD1*-marked and terminal cytotoxic states.

Overall, these findings support an exploratory model in which psoriasis is associated with cytotoxic-program-biased remodeling of circulating γδ T cells and reduced peripheral representation of *CSMD1*-marked cells. The stronger *CSMD1*-associated signal in psoriatic skin further supports the presence of blood–skin compartmental differences involving *CSMD1*-associated γδ T-cell programs.

## 4 Discussion

Single-cell sequencing has provided an increasingly powerful framework for resolving immune-cell heterogeneity and disease-associated remodeling at cellular resolution. In psoriasis, γδ T cells have been recognized as important contributors to inflammatory responses, particularly through IL-17-producing and tissue-associated programs (4, 13, 14, 16). However, most previous studies have focused on γδ T cells in psoriatic skin, tissue-resident γδ T-cell responses, or broader immune-cell interactions within lesional microenvironments (4–6, 13, 14). Whether psoriasis also reshapes the circulating γδ T-cell compartment, and whether such remodeling reflects the emergence of new disease-specific populations or redistribution of pre-existing states, has remained less clear.

In this study, we addressed this question by constructing a state-resolved single-cell map of peripheral blood γδ T cells from healthy donors and patients with psoriasis. Rather than revealing an overt psoriasis-specific γδ T-cell population, our data support a different model: psoriasis remodels the balance and functional organization of pre-existing circulating γδ T-cell states. This remodeling was reflected by three related features: relative enrichment of effector-associated transcriptional states, reduced peripheral representation of a *CSMD1*-marked higher-pseudotime state, and transcriptome-inferred changes in communication- and regulatory-associated patterns. Notably, this peripheral blood dataset did not reveal a clear γδ17-like transcriptional state. *IL17A*, *IL17F*, *IL23R*, *CCR6*, *RORC*, *CCR2*, *CXCR6*, and related γδ17-associated markers were absent or detected only at very low levels, indicating that the dominant circulating γδ T-cell changes captured here were cytotoxicity, activation, and *CSMD1*-associated rather than IL-17-associated. Thus, peripheral γδ T-cell remodeling in psoriasis appears to be less a matter of generating a new cell type than of redirecting an existing differentiation continuum toward effector polarization.

One notable observation in our dataset was the reduced peripheral representation of the *CSMD1*-marked γδ T-cell state in psoriasis. This finding should be interpreted in the context of the Monocle2-inferred transcriptional continuum. Although psoriasis-derived γδ T cells were relatively enriched in higher-pseudotime, cytotoxicity-associated regions, *CSMD1*-marked cells occupied a more restricted higher-pseudotime region and showed a higher-pseudotime-associated pattern of *CSMD1* expression. Thus, within the resolution of this cross-sectional dataset, the *CSMD1*-marked state was not resolved as a broad transitional population or an independent lineage, but rather as a distinct transcriptional state associated with a restricted activation/cytotoxicity-related region. Importantly, the *CSMD1*-marked γδ T-cell state should not be interpreted as an IL-17-producing γδ T-cell population. This state did not show enrichment of γδ17-associated genes, including *IL17A*, *IL17F*, *IL23R*, *CCR6*, *RORC*, *CCR2*, or *CXCR6*, supporting its distinction from a classical γδ17-like program in the present peripheral blood dataset. Together, these findings suggest that psoriasis-associated γδ T-cell remodeling is not a uniform expansion of all higher-pseudotime states. Instead, cytotoxic and NK-like effector-associated programs were relatively enriched in circulation, whereas the *CSMD1*-marked higher-pseudotime state showed reduced peripheral representation.

These observations raise the possibility that the reduced peripheral representation of the *CSMD1*-marked γδ T-cell state may be related to compartment-specific γδ T-cell organization in psoriasis. In our tissue-level mIF analysis, psoriatic lesions showed increased TCRδ-, GZMK-, and CSMD1-associated signals, and segmentation-based quantification supported the presence of TCRδ^+^CSMD1^+^GZMK^+^ cells in lesional skin (**Supplementary Figure 13**). However, the skin samples were not paired with the PBMC scRNA-seq samples, and the mIF data do not establish that lesional TCRδ^+^CSMD1^+^GZMK^+^ cells are clonally or developmentally related to the circulating CSMD1-marked γδ T-cell state. External PBMC and skin single-cell datasets provided additional contextual information for evaluating γδ T-cell and *CSMD1* transcript detection across circulating and tissue compartments (5, 6). However, because these datasets were not γδ T cell-enriched and *CSMD1* transcripts were sparsely detected, analyses centered on *CSMD1*-expressing γδ T cells should be interpreted as exploratory rather than as independent evidence for *CSMD1*-associated communication networks. Together with previous studies showing that γδ T cells can traffic dynamically during psoriasis-like skin inflammation and that psoriasis involves systemic immune alterations beyond lesional skin (13, 29), our findings are compatible with possible blood–skin compartmental differences involving *CSMD1*-associated γδ T-cell programs.

Although the *CSMD1*-marked state provides a useful focal point for considering blood–skin compartmental differences, it represents only one component of the broader psoriasis-associated remodeling of circulating γδ T cells. Beyond this state, the overall trajectory structure suggests effector-skewed remodeling of the circulating γδ T-cell compartment in psoriasis. Along the inferred transcriptional continuum, γδ T cells were ordered from Naive-like and activation-associated states toward Cytotoxic effector, NK-like, Cytotoxic memory-like, and Terminal-branch states. This transition was accompanied by increased expression of cytotoxic and migratory genes, including *ZEB2*, *GNLY*, *GZMB*, *GZMH*, *FGFBP2*, *CX3CR1*, and *FCGR3A*. The involvement of *ZEB2* is consistent with previous studies linking *ZEB2* to terminal cytotoxic T-cell differentiation (30, 31). In line with previous work showing that γδ T cells can acquire diverse effector and tissue-adapted programs under inflammatory conditions (7, 8, 13, 14), our findings suggest that psoriasis-associated γδ T-cell remodeling is not confined to lesional skin, but may reflect a systemic bias toward effector polarization. Whether these terminal cytotoxic-associated states actively contribute to tissue inflammation or instead represent a consequence of chronic immune activation remains to be determined.

Transcriptome-inferred communication analyses provided an additional layer for evaluating state-associated organization of circulating γδ T cells. The γδ T-cell-restricted CellChat network indicated that inferred sender and receiver roles varied across annotated states: Cytotoxic-associated tended to act more as senders, whereas Naive-like and activation-associated states remained relatively receiver-biased. These findings place known immune-recognition and inflammatory pathways, including LLT1–CD161 (9, 32, 33), HLA-E–CD94/NKG2 (10, 34, 35), and MIF-associated receptor systems (11, 12, 36, 37), into a state-resolved sender–receiver framework in psoriasis. The *CSMD1*-marked γδ T-cell state did not emerge as a dominant hub within the intra-γδ T-cell network. External PBMC and skin datasets provided additional tissue-context information, but because these datasets were not γδ T cell-enriched and *CSMD1* transcripts were sparsely detected, *CSMD1*-centered heterotypic communication analyses should be interpreted as exploratory rather than definitive.

Metabolite–sensor communication analysis provided a complementary transcriptome-inferred layer. Rather than forming a separate metabolic story, the mCCC results converged with the trajectory and ligand–receptor analyses: communication became concentrated around activated-to-terminal effector interactions. Transitional activated and Activation-regulated γδ T cells contributed prominently as inferred sender states, whereas terminal effector-related states were more frequently positioned as receivers. Several candidate metabolite–sensor or metabolite–transporter axes, including D-mannose–SLC2A3, cholesterol–RORA, IP_3_–ITPR1/2, phosphate–SLC20A2, and choline- or GABA-related routes, contributed to this inferred network. Thus, mCCC analysis provides a useful framework for prioritizing candidate metabolite–sensor communication events from scRNA-seq data (38). This interpretation is also consistent with broader evidence that nutrient availability, Ca²^+^ signaling, IP_3_ receptor-dependent homeostasis, and mitochondrial redox programs can shape lymphocyte activation and immune-state remodeling (15, 22, 23, 39–42). The phosphate-related route is notable given prior links between phosphate transporter expression and lymphocyte differentiation states (21). Thus, altered metabolic sensing and transport may represent one mechanism by which the effector-skewed γδ T-cell network is stabilized in psoriasis, although this possibility will require direct metabolomic and perturbation-based validation.

Regulatory analyses provide a possible transcriptional explanation for this state-associated remodeling. FOXP1 was associated with early-state maintenance, consistent with its reported role in maintaining naive T-cell quiescence (25, 26). STAT1 was linked to the activation-to-late transition, consistent with the role of STAT1 signaling in T-cell activation and differentiation programs (27). NFATC3 was associated with terminal cytotoxic-associated states, in line with previous evidence that NFATC3 regulates genes involved in T-cell activation (28). These regulators did not point to a single master switch. Instead, they suggest a layered regulatory architecture in which early-state maintenance, transitional restraint, and terminal effector stabilization are separable features of γδ T-cell remodeling. This interpretation is also compatible with TCF1/LEF1-centered networks preserving less differentiated T-cell states (43–45) and *ZEB2* promoting terminal cytotoxic differentiation (30, 31). In this view, psoriasis-associated effector skewing may reflect differential representation of early-like, transitional, and cytotoxic-associated regulatory programs rather than induction of an entirely disease-exclusive transcriptional state.

More broadly, these findings have several implications. First, peripheral γδ T-cell remodeling in psoriasis may be better understood as part of systemic immune-state reorganization rather than as the *de novo* generation of a disease-exclusive cell population (29). Second, the *CSMD1*-marked state nominates a candidate higher-pseudotime γδ T-cell state for future blood–skin validation. Third, state-resolved communication features, including cytotoxic sender-biased ligand–receptor patterns, exploratory *CSMD*1-associated tissue-context analyses, and candidate metabolite–sensor axes, may provide hypothesis-generating readouts of γδ T-cell remodeling in psoriasis. Whether these features can complement existing therapeutic frameworks targeting the IL-23/IL-17 axis, or whether they may provide biomarkers for disease activity, tissue involvement, or treatment response, remains an important question for future investigation (1–3, 46–49).

Several limitations should be acknowledged. The cohort size was small, which limits the ability to resolve inter-individual heterogeneity and to generalize subtle compositional differences. In addition, the communication, mCCC, regulon, and in silico perturbation analyses are computational inferences and should therefore be interpreted as hypothesis-generating rather than definitive evidence of molecular mechanism. Finally, the proposed blood–skin redistribution of the *CSMD1*^+^ γδ T cell state remains a possible interpretation of the current findings. Although the lower abundance of this state in peripheral blood and the increased *CSMD1*-associated signals in psoriatic skin is compatible with such a model, they do not demonstrate cellular migration, tissue recruitment, or clonal continuity. Because the blood and skin samples were not paired and the study was cross-sectional, direct conclusions regarding redistribution or causality cannot be drawn. Future studies using larger cohorts, paired blood and skin samples, longitudinal sampling, TCR clonotype tracking, functional migration assays, and direct perturbation experiments will be required to test these hypotheses.

In summary, our study provides a state-resolved framework of circulating γδ T-cell remodeling in psoriasis. We observe reduced peripheral representation of a *CSMD1*-marked higher-pseudotime γδ T-cell state, alongside enrichment of cytotoxic/NK-like effector-associated programs. By integrating cell-state composition, Monocle2-inferred transcriptional ordering, transcriptome-based ligand–receptor interactions, candidate metabolite–sensor communication, and regulon activity, we establish a systems-level framework to prioritize γδ T-cell states, pathways, and regulatory programs for future validation. These findings offer a foundation for exploring how circulating γδ T-cell changes may relate to systemic immune alterations and tissue-associated inflammation in psoriasis.

## Data Availability

The data presented in this study are deposited in the Gene Expression Omnibus (GEO) repository, accession number GSE335977. The analysis scripts used in this study are available at GitHub: https://github.com/cshinjnu-eng/scRNA-seq-gdT-psoriasis.
