# Correction: Peripheral γδ T-cell remodeling in psoriasis is associated with effector-biased transcriptional organization and reduced representation of a circulating *CSMD1*-marked state

**DOI:** 10.3389/fimmu.2026.1974909

**Published:** 2026-10-06

**Authors:** Nanxi Shi, Yiming Chen, Jiale Tang, Sihan Chen, Xi Lin, Yingying Ye, Jingsong Zhang, Ruili Chen, Jiaqi Zeng, Guodong Sun, Xiaoqiao Yang, Suhua Zhong, Fang Huang, Zhenhua Li, Xin Tang, Shi Wu, Yunting Liang

**Affiliations:** 1 The Biomedical Translational Research Institute, Key Laboratory of Ministry of Education for Viral Pathogenesis & Infection Prevention and Control, School of Life Science and Technology, Jinan University, Guangzhou, China; 2 The Biomedical Translational Research Institute, Key Laboratory of Ministry of Education for Viral Pathogenesis & Infection Prevention and Control, School of Medicine, Jinan University, Guangzhou, China; 3 School of Life Science and Biopharmaceutics, Guangdong Pharmaceutical University, Guangzhou, China; 4 Department of Systems Biomedical Sciences, School of Medicine, Jinan University, Guangzhou, China; 5 Institute of Ecological Sciences, School of Life Sciences, South China Normal University, Guangzhou, China; 6 Zhuhai People’s Hospital (The Affiliated Hospital of Beijing Institute of Technology, Zhuhai Clinical Medical College of Jinan University), Jinan University, Zhuhai, China; 7 Guangdong Provincial Key Laboratory of Spine and Spinal Cord Reconstruction, The Fifth Affiliated Hospital of Jinan University, Heyuan, China; 8 Department of Gastroenterology, The Affiliated Guangdong Second Provincial General Hospital of Jinan University, Guangzhou, China; 9 Department of Computer Science, Guangdong Institute of Smart Education, Jinan University, Guangzhou, China; 10 Department of Dermatology, The First Affiliated Hospital of Jinan University, Guangzhou, China

**Keywords:** cell heterogeneity, peripheral blood, psoriasis, scRNA-seq, γδ T cell

Author “Shi Wu” was erroneously omitted as corresponding author. The correct corresponding author(s) are “Yunting Liang, Shi Wu, Xin Tang, and Zhenhua Li”.

The original version of this article has been updated.

