# Supplementary material for: Peripheral γδ T-cell remodeling in psoriasis is associated with effector-biased transcriptional organization and reduced representation of a circulating CSMD1-marked state
Source: Front Immunol. 2026 Aug 20;17:1885325. doi: 10.3389/fimmu.2026.1885325 (PMC13538830; doi:10.3389/fimmu.2026.1885325)
Supplement: Supplementary file 1 [file DataSheet1.pdf]

Supplementary Materials

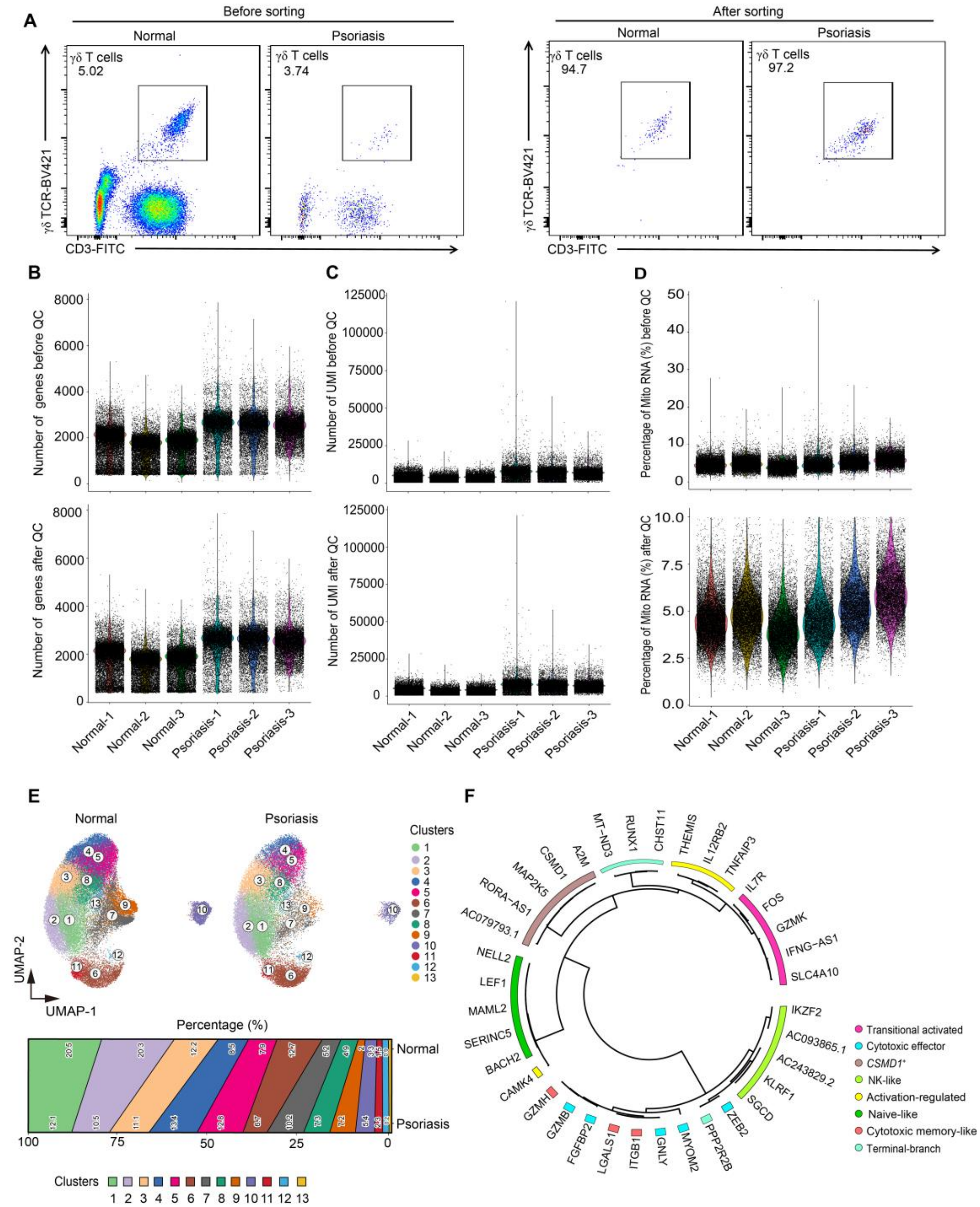

Supplementary Figure 1. γδ T-cell sorting, quality control, initial clustering and marker-based state annotation. (A) Representative flow cytometry plots showing enrichment of peripheral blood γδ T cells before and after sorting in the normal and psoriasis groups. Cells were gated as $CD3^{+}$ $TCR\gamma\delta^{+}$. (B–D) Quality control metrics shown for individual samples before and after filtering. (B) Number of detected genes.

(C) Number of UMIs. (D) Percentage of mitochondrial RNA. (E) UMAP visualization of the integrated dataset showing the initial unsupervised clustering of γδ T cells into 13 clusters, displayed separately for the normal and psoriasis groups. The stacked bar plot shows the relative proportion of each cluster in the two groups. (F) Circular plot showing representative marker genes supporting the annotation of circulating γδ T-cell states. Colors indicate the eight annotated γδ T-cell states: Transitional activated, Cytotoxic effector, *CSMD1*$^{+}$, NK-like, Activation-regulated, Naive-like, Cytotoxic memory-like, and Terminal-branch γδ T cells.

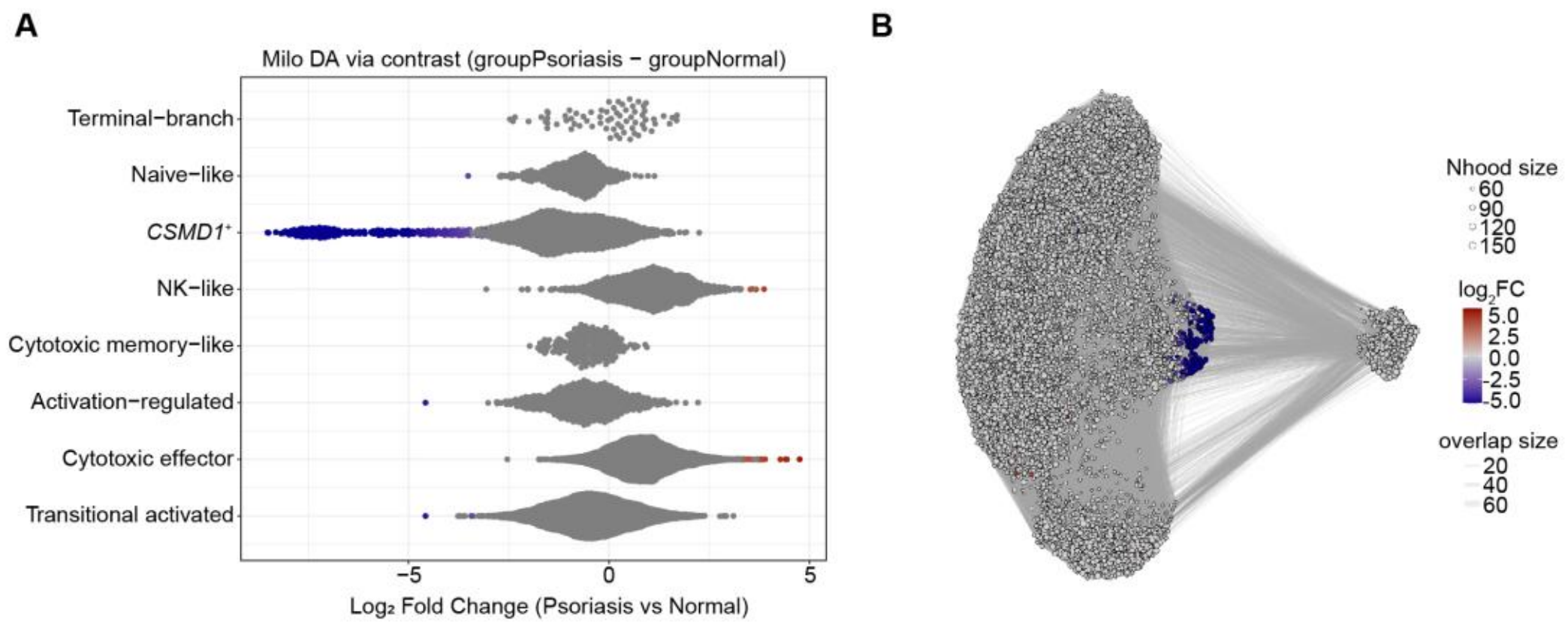

Supplementary Figure 2. Milo neighbourhood differential abundance analysis of γδ T-cell states. (A) Milo beeswarm plot showing neighbourhood-level abundance changes across annotated γδ T-cell states using Psoriasis versus Normal as the contrast. Negative $\log_2$ fold changes indicate lower abundance in psoriasis, whereas positive values indicate higher abundance. *CSMD1*$^+$ neighbourhoods were predominantly shifted toward lower abundance, while cytotoxic effector- and NK-like-associated neighbourhoods showed relative positive shifts. (B) Milo neighbourhood graph showing the distribution of differentially abundant neighbourhoods on the γδ T-cell manifold. Node size denotes neighbourhood size, color denotes $\log_2$ fold change, and edge thickness denotes neighbourhood overlap. Milo was used as a donor-aware sensitivity analysis given the limited cohort size.

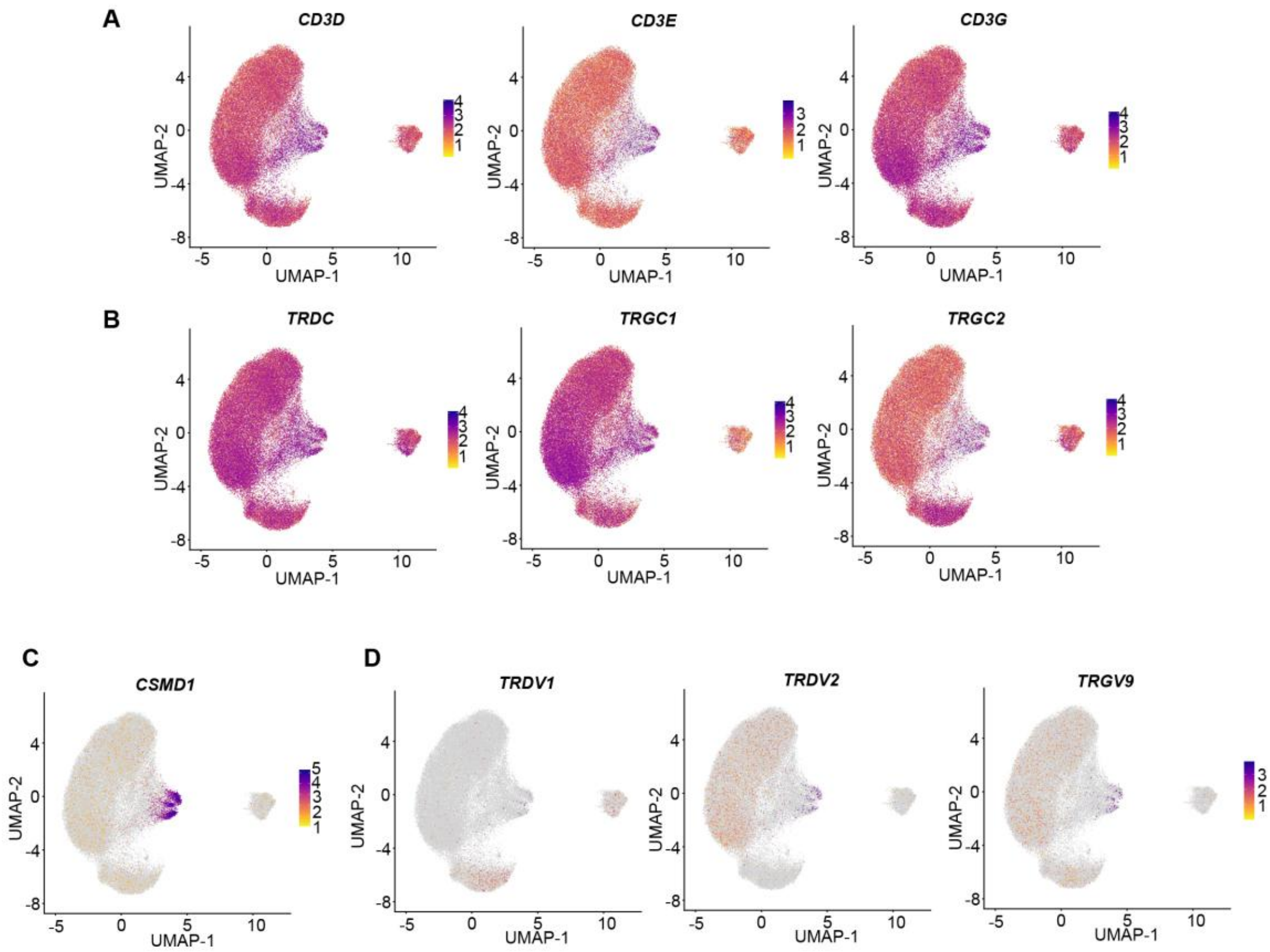

Supplementary Figure 3. Co-expression assessment of *CSMD1* with core T-cell and γδ TCR-associated genes. FeaturePlot analysis was performed to evaluate whether CSMD1-associated signals were located within the γδ T-cell transcriptional compartment. (A) Core T-cell markers, including *CD3D*, *CD3E*, and *CD3G*, were visualized across the integrated γδ T-cell UMAP. (B) Core γδ TCR-associated genes, including *TRDC*, *TRGC1*, and *TRGC2*, were examined to assess γδ T-cell identity. (C) *CSMD1* expression was visualized to determine the localization of *CSMD1*-associated cells relative to the γδ T-cell manifold. (D) Exploratory TCR variable-region transcripts, including *TRDV1, TRDV2*, and *TRGV9*, were examined as transcript-level indicators of Vδ/Vγ usage.

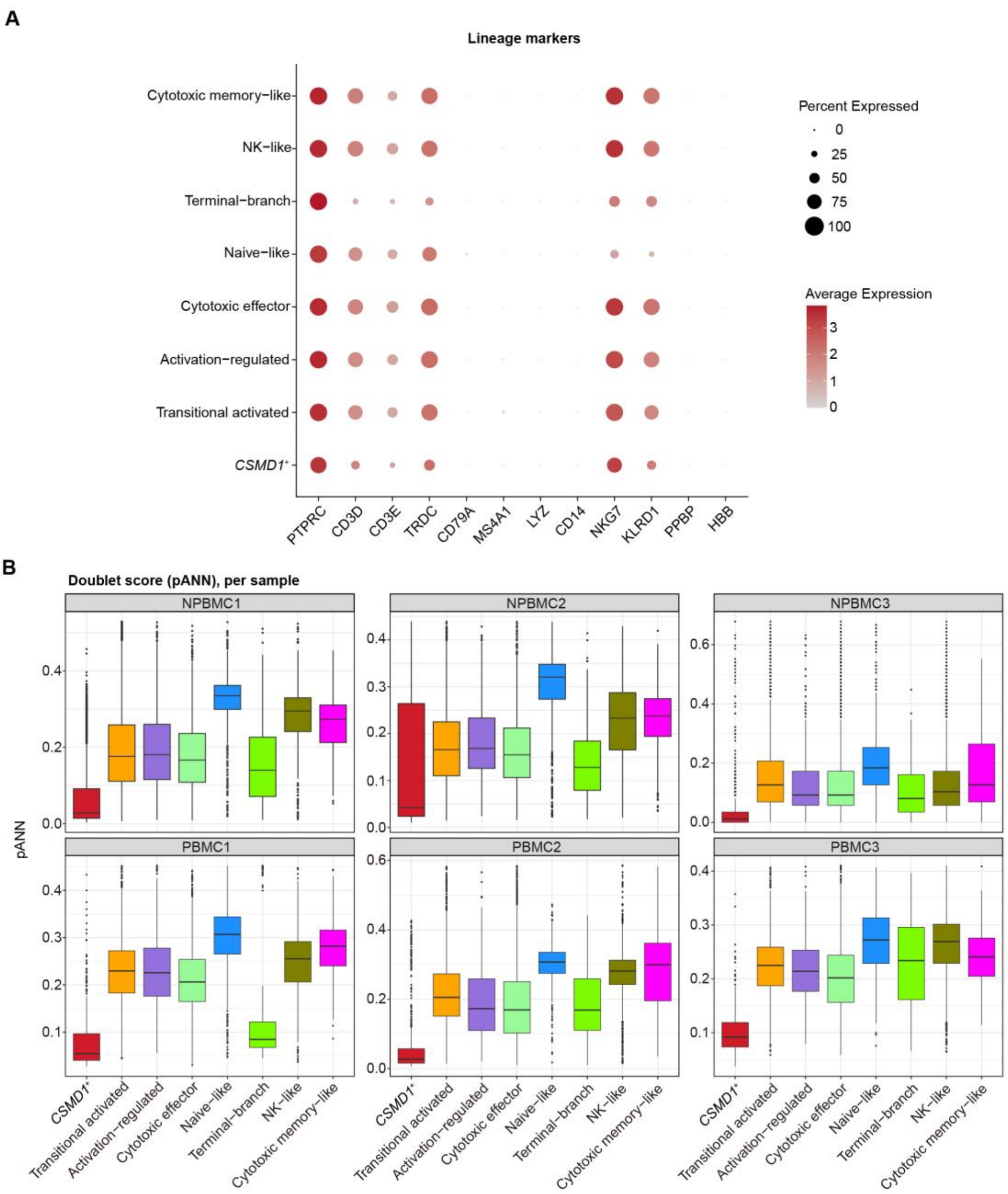

Supplementary Figure 4. Lineage-marker and doublet-score assessment of annotated γδ T-cell states. Representative T-cell/γδ T-cell markers and non-T-cell lineage markers were assessed across annotated γδ T-cell states. (A) Dot plot of representative lineage and contamination-associated markers across γδ T-cell states. Dot size indicates the percentage of expressing cells, and color intensity reflects average expression. All states expressed T-cell/γδ T-cell markers (PTPRC, CD3D, CD3E, TRDC), while B-cell, myeloid, platelet, and erythroid markers (CD79A, MS4A1, LYZ, CD14, PPBP, HBB) were low or absent. Cytotoxic markers (NKG7, KLRD1) were enriched in cytotoxic/NK-like states. (B) Distribution of DoubletFinder-derived pANN

scores across γδ T-cell states in each donor (NPBMC1–3: Normal; PBMC1–3: Psoriasis). The CSMD1-marked state did not show elevated doublet scores, indicating it is unlikely driven by doublet contamination. These analyses support lineage identity and minimal technical contamination after quality control and doublet filtering.

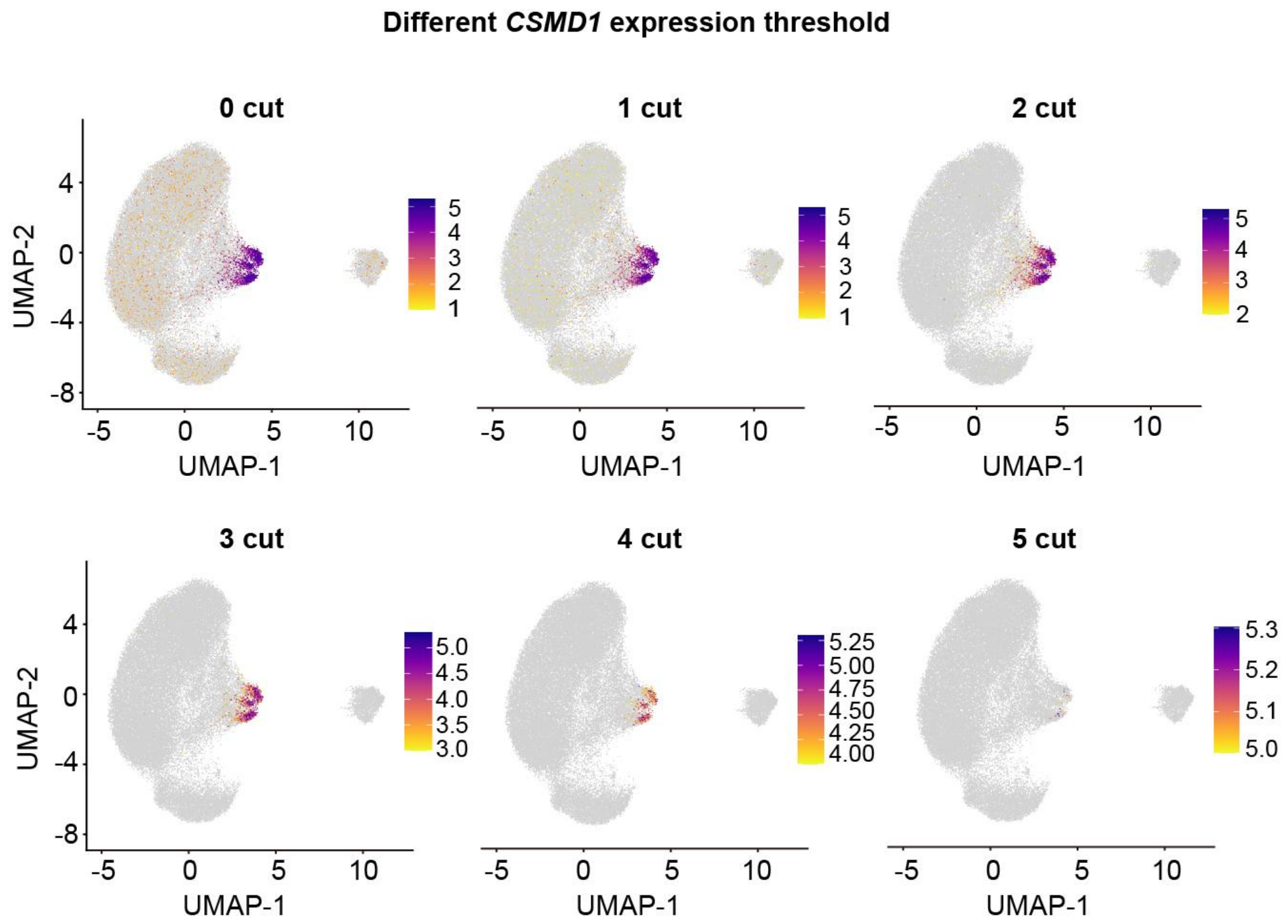

Supplementary Figure 5. Sensitivity analysis using different CSMD1 expression thresholds. UMAP visualization of CSMD1-expressing cells under increasing CSMD1 expression thresholds. Low-threshold CSMD1 detection included sparse signals across the γδ T-cell manifold, whereas higher-threshold CSMD1-expressing cells remained concentrated in a restricted region corresponding to the CSMD1-marked γδ T-cell state. This analysis supports state-restricted enrichment of higher CSMD1 signal across different threshold settings, while the sparse nature of CSMD1 detection requires cautious interpretation.

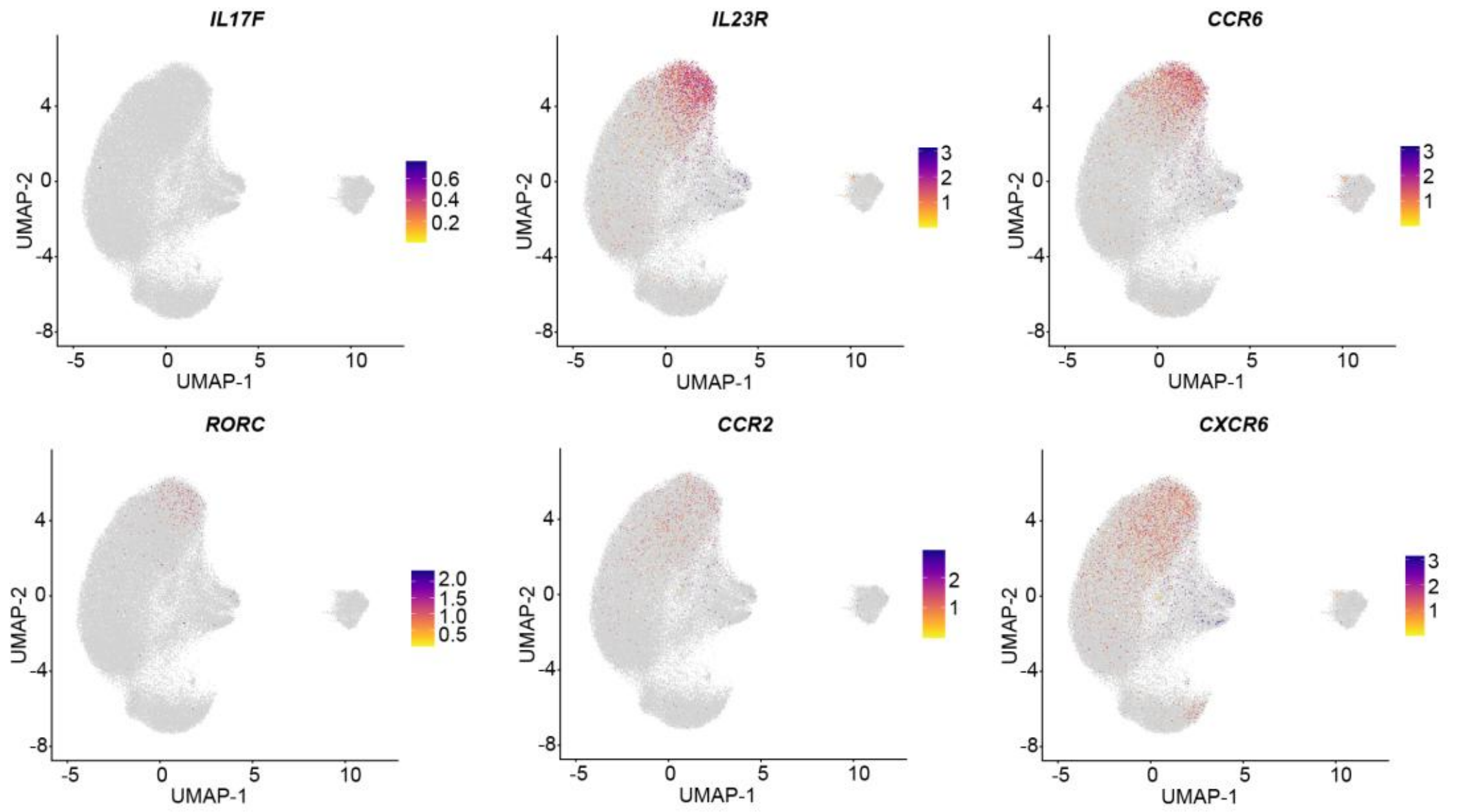

Supplementary Figure 6. Expression of γδ17-associated markers in peripheral blood γδ T cells. FeaturePlots showing γδ17-associated marker expression in FACS-enriched peripheral blood γδ T cells. *IL17A* was not detected in retained γδ T cells based on raw UMI counts. IL17F showed minimal expression, whereas *IL23R*, *CCR6*, *RORC*, *CCR2*, and *CXCR6* were detected at low or sparse levels. No discrete $IL17A^{+}/IL17F^{+}/RORC^{+}$ γδ17-like state was identified in this peripheral blood dataset.

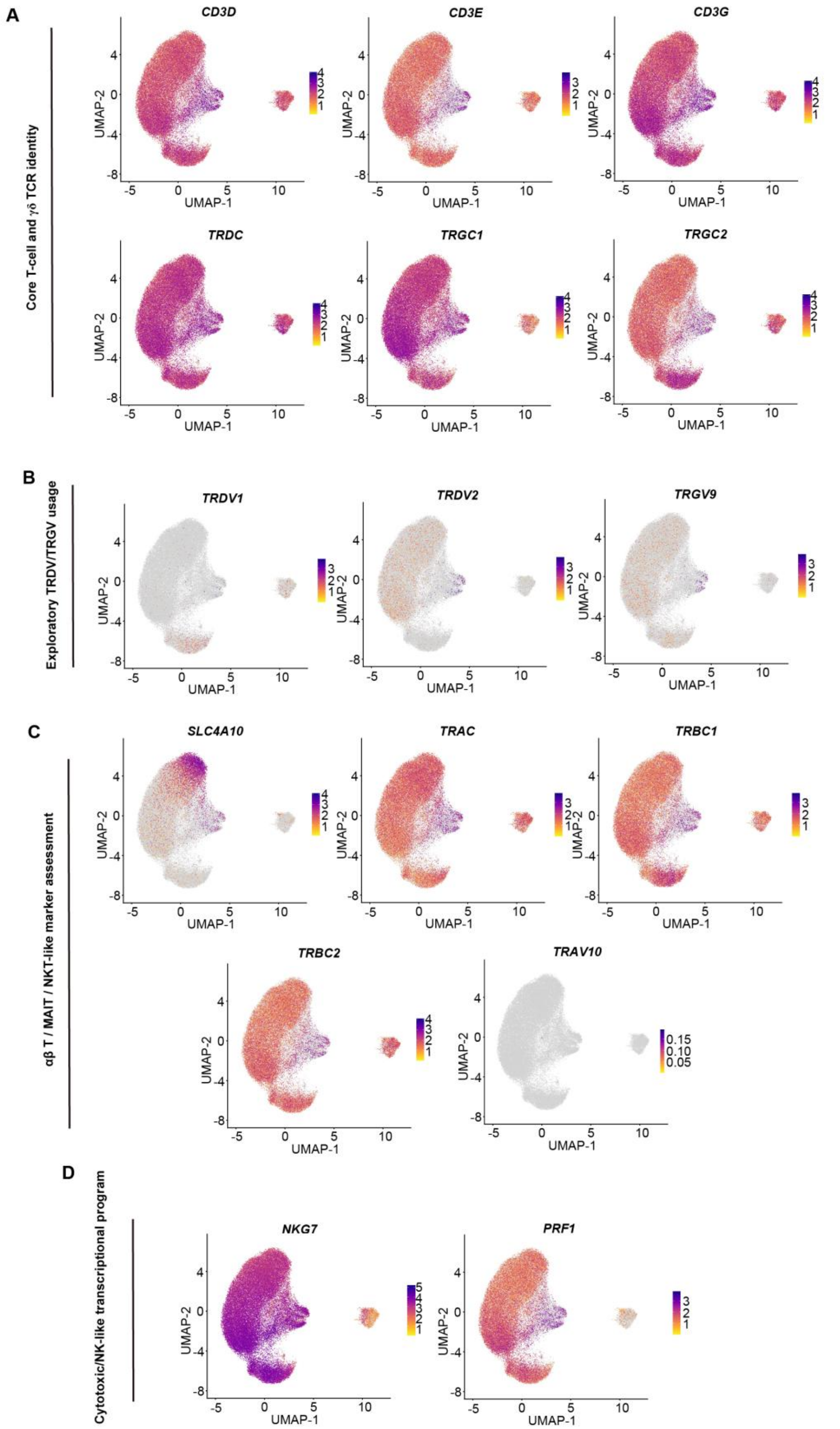

Supplementary Figure 7. TCR gene usage and lineage-associated marker assessment across annotated γδ T-cell states. (A) Core T-cell and γδ TCR markers

(*CD3D*, *CD3E*, *CD3G*, *TRDC*, *TRGC1*, and *TRGC2*). (B) Exploratory TRDV/TRGV usage markers (*TRDV1*, *TRDV2*, and *TRGV9*). (C) αβ T/MAIT/NKT-like marker assessment (*SLC4A10*, *TRAC*, *TRBC1*, *TRBC2*, and *TRAV10*). (D) Cytotoxic/NK-like transcriptional program markers (*NKG7* and *PRF1*).

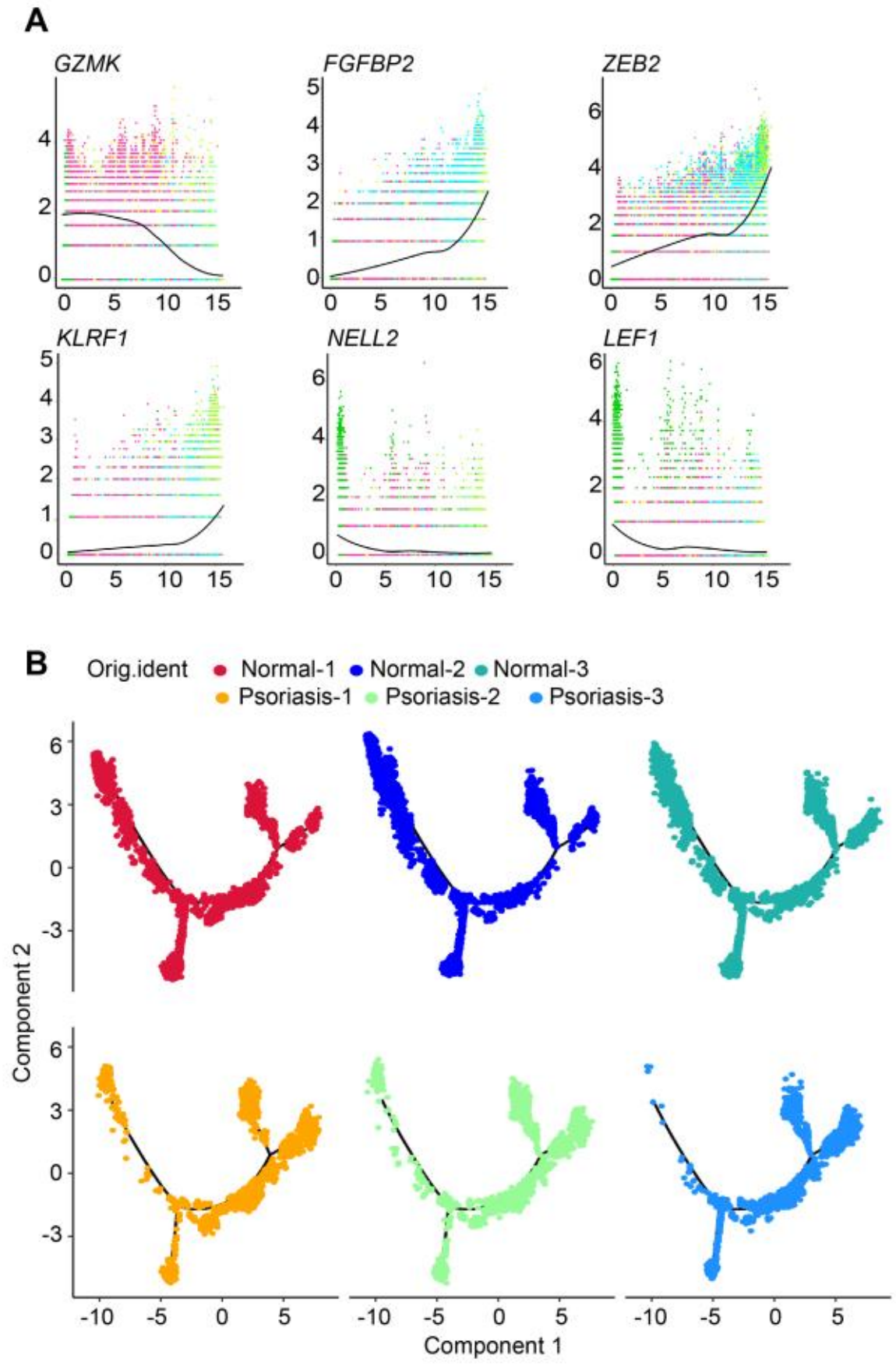

Supplementary Figure 8. Dynamic changes of key marker genes along the γδ T-cell pseudotime trajectory and distribution of individual samples across the trajectory. (A) Expression dynamics of representative genes along pseudotime. Early/transitional markers, including *GZMK*, *NELL2* and *LEF1*, and late cytotoxic-associated markers, including *FGFBP2*, *ZEB2* and *KLRF1*, are shown. Dots represent single cells, and black curves indicate the fitted overall trend. (B) Distribution of cells from individual donors in the trajectory space (Component 1/2), grouped by sample origin (Orig.ident). The upper panels show Normal-1/2/3, and the lower panels show Psoriasis-1/2/3. Black lines indicate the inferred trajectory backbone.

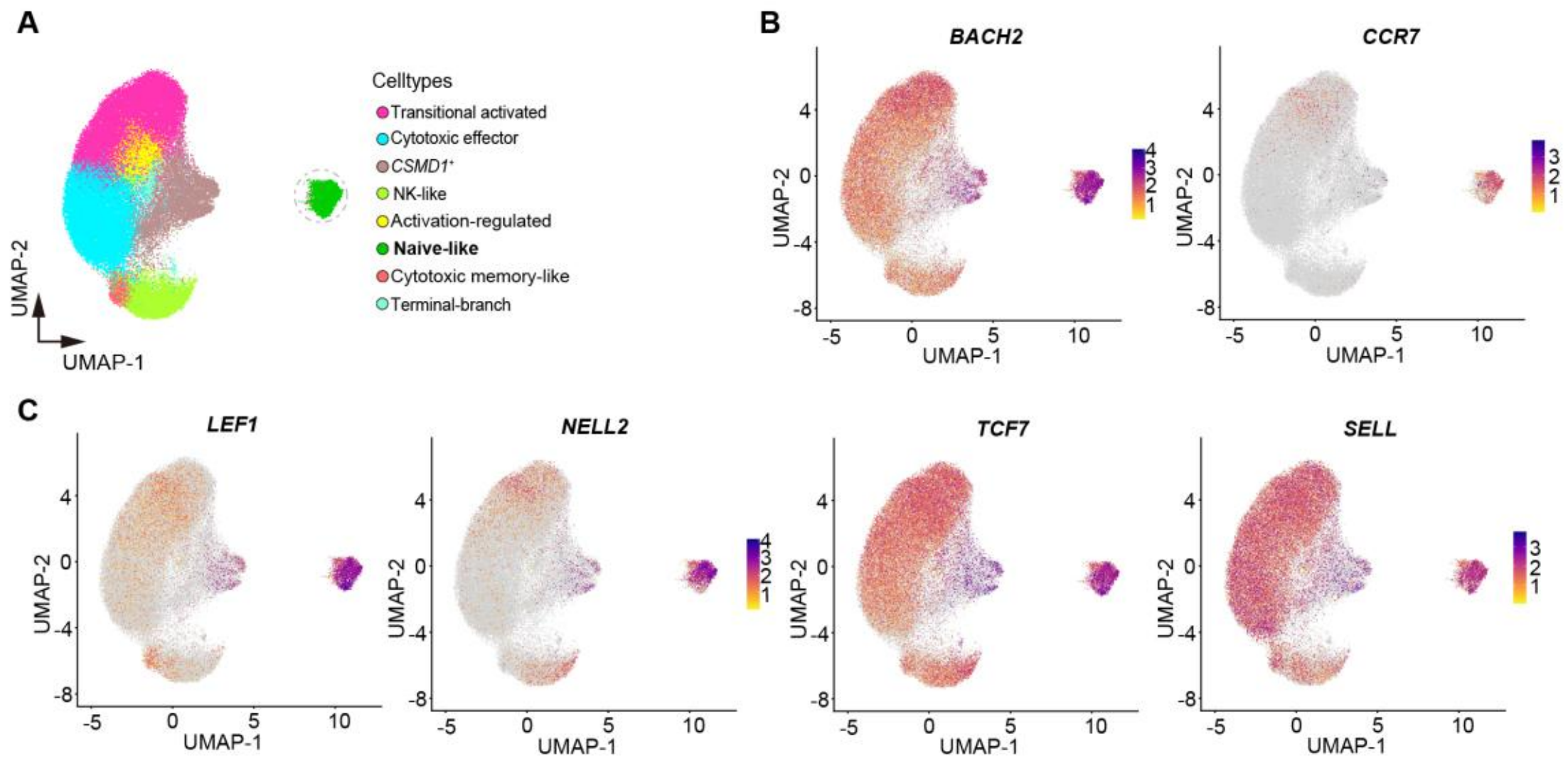

Supplementary Figure 9. Marker-based support for root-state assignment in the Monocle2-inferred γδ T-cell transcriptional continuum. (A) UMAP visualization of annotated γδ T-cell states, showing the spatial position of the Naive-like γδ T-cell state within the integrated γδ T-cell manifold. (B–C) Overall UMAP FeaturePlot analysis of early/naive-associated genes, including BACH2, *CCR7*, *LEF1*, *NELL2*, *TCF7*, and *SELL*. These markers were enriched in the Naive-like γδ T-cell region and partially retained in adjacent lower-pseudotime/activation-associated regions, whereas cytotoxic/NK-like and terminal-associated regions showed lower expression. Together with the lower-pseudotime positioning of Naive-like γδ T cells and their higher CytoTRACE-predicted differentiation potential, these marker-expression patterns supported the assignment of the Naive-like state as the root for trajectory orientation. The trajectory was interpreted as an inferred transcriptional continuum rather than a validated lineage hierarchy.

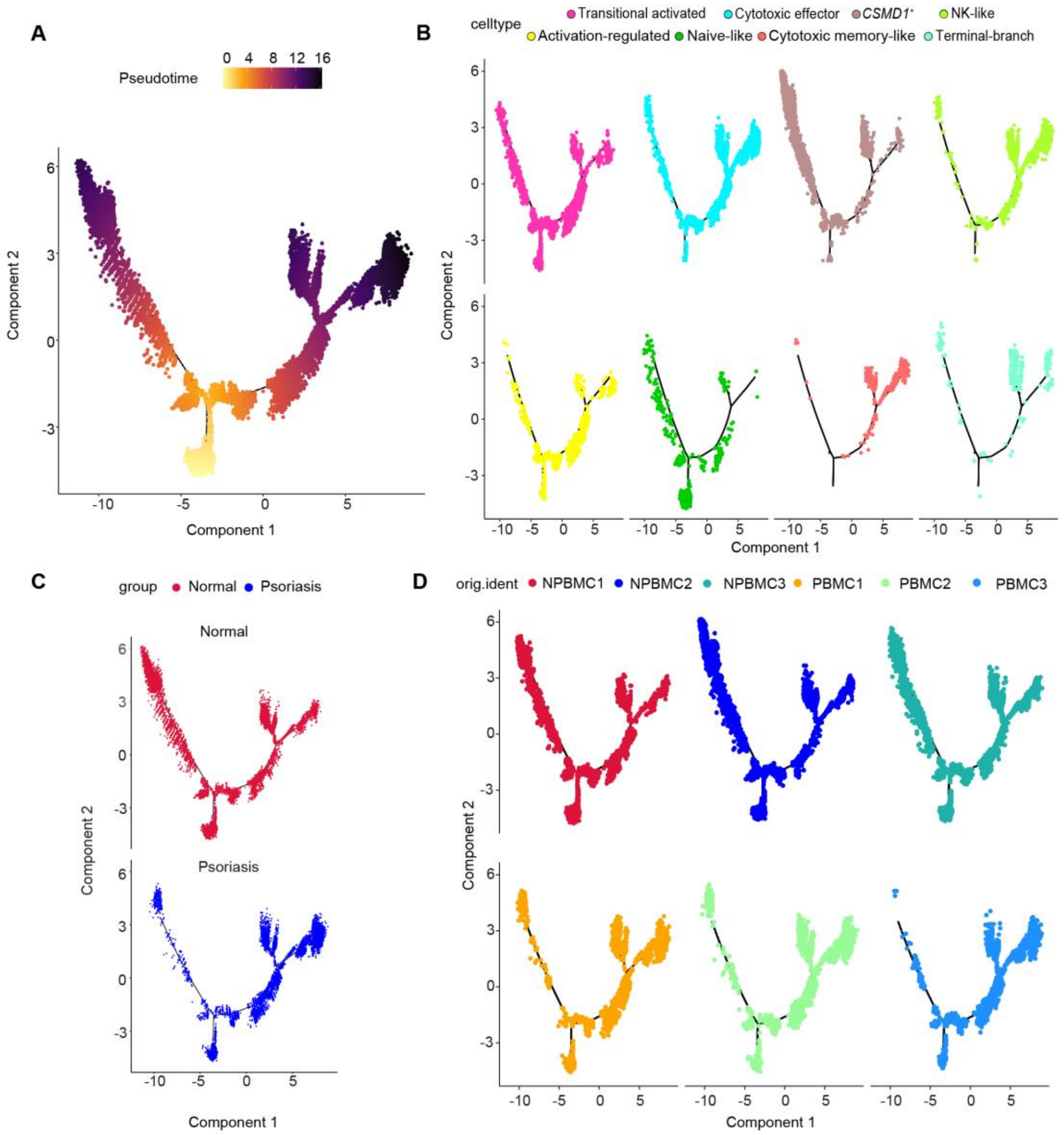

Supplementary Figure 10. Ordering-gene sensitivity analysis after excluding CSMD1 and major state-defining marker genes. Monocle2 trajectory inference was repeated after excluding the top three marker genes from each annotated γδ T-cell state before ordering-gene selection. Because *CSMD1* was among the top three marker genes of the *CSMD1*-marked state, it was also excluded in this analysis. Ordering genes were then reselected using the same q-value-based criterion, and the trajectory was reconstructed using the same Monocle2 workflow. (A) Reconstructed Monocle2 trajectory colored by inferred pseudotime, showing a continuous pseudotime gradient after marker-gene exclusion. (B) Reconstructed trajectory split by annotated γδ T-cell

state, showing that the major γδ T-cell states retained broadly comparable positions along the inferred continuum. (C) Reconstructed trajectory split by group, showing the distribution of Normal and psoriasis cells along the marker-excluded trajectory. (D) Reconstructed trajectory split by individual donor, showing that the major trajectory structure was retained across samples.

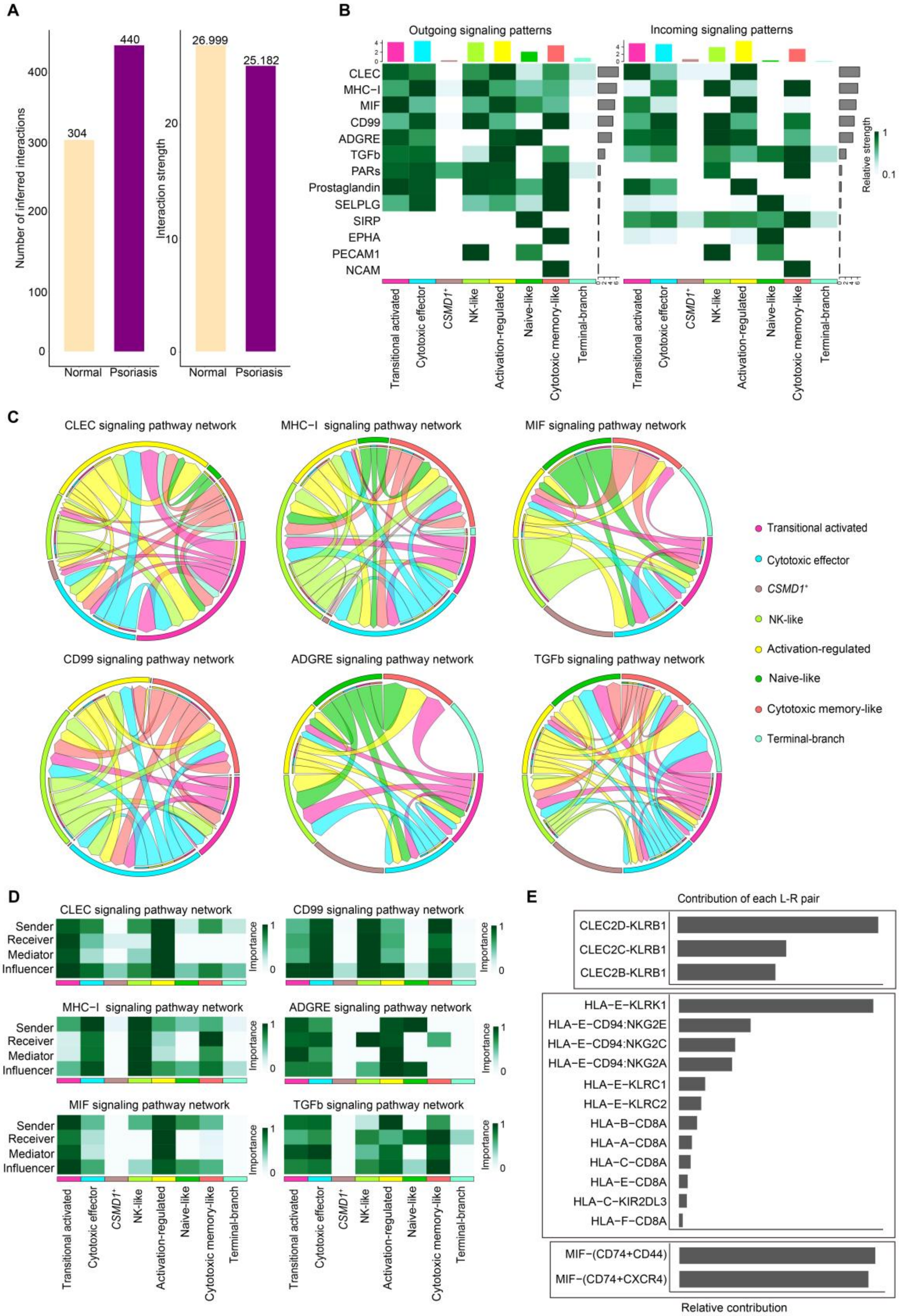

Supplementary Figure 11. Enhanced inter-subset communication among γδ T cells in psoriasis and contribution analysis of key signaling pathways and ligand–receptor axes. (A) Summary of inferred γδ T–γδ T communication networks in Normal

and Psoriasis samples. The left panel shows the number of inferred interactions, and the right panel shows the overall interaction strength. Psoriasis samples showed an increased number of inferred interactions but a modest reduction in overall interaction strength. (B) Heat maps showing pathway-level outgoing and incoming signaling patterns across annotated γδ T-cell states. Color intensity indicates relative signaling strength. Columns represent Transitional activated, Cytotoxic effector, CSMD1^{+}, NK-like, Activation-regulated, Naive-like, Cytotoxic memory-like, and Terminal-branch γδ T-cell states. Rows indicate signaling pathways inferred by CellChat. (C) Chord diagrams showing representative pathway-specific γδ T–γδ T communication networks, including CLEC, MHC-I, MIF, CD99, ADGRE, and TGF-β signaling. Edge width indicates inferred information flow, and colors denote sender γδ T-cell states. (D) Pathway-level role analysis showing the relative importance of each γδ T-cell state as sender, receiver, mediator, and influencer across representative signaling pathways. (E) Relative contribution of major ligand–receptor pairs within selected signaling pathways, including CLEC2 family–KLRB1, HLA-related receptor pairs, and MIF–(CD74+CD44/CXCR4) interactions.

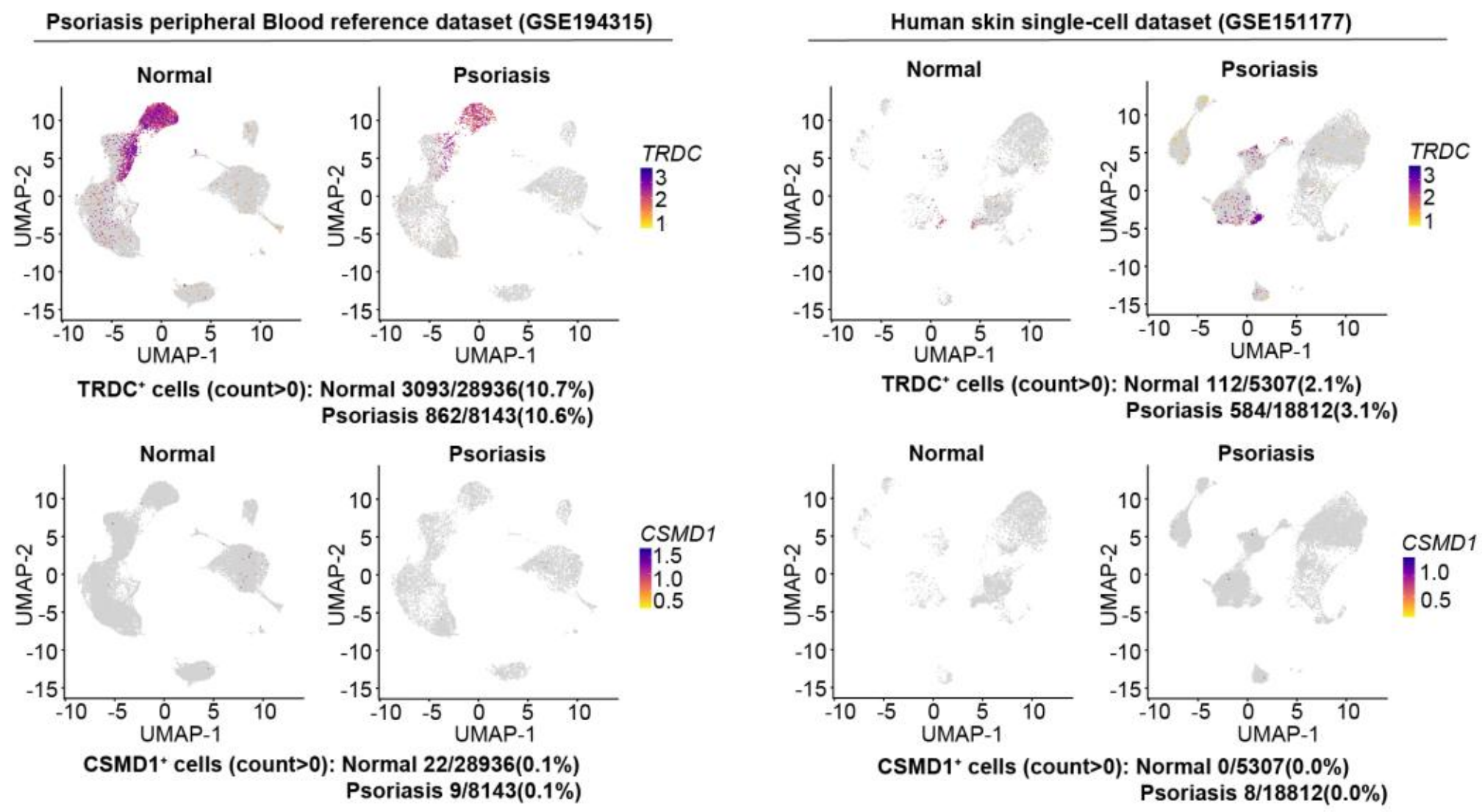

Supplementary Figure 12. Sparse CSMD1 detection in external non-enriched PBMC and skin single-cell datasets. FeaturePlots show *TRDC* and *CSMD1* expression in the external PBMC dataset GSE194315 and skin dataset GSE151177. *TRDC*-positive cells were detectable in both datasets, whereas *CSMD1* expression was sparse. Because these public datasets were not γδ T cell-enriched and *CSMD1* detection was limited, analyses centered on *CSMD1*-expressing γδ T cells were interpreted as exploratory transcriptomic context rather than independent validation of a *CSMD1*-marked γδ T-cell state or evidence of tissue trafficking.

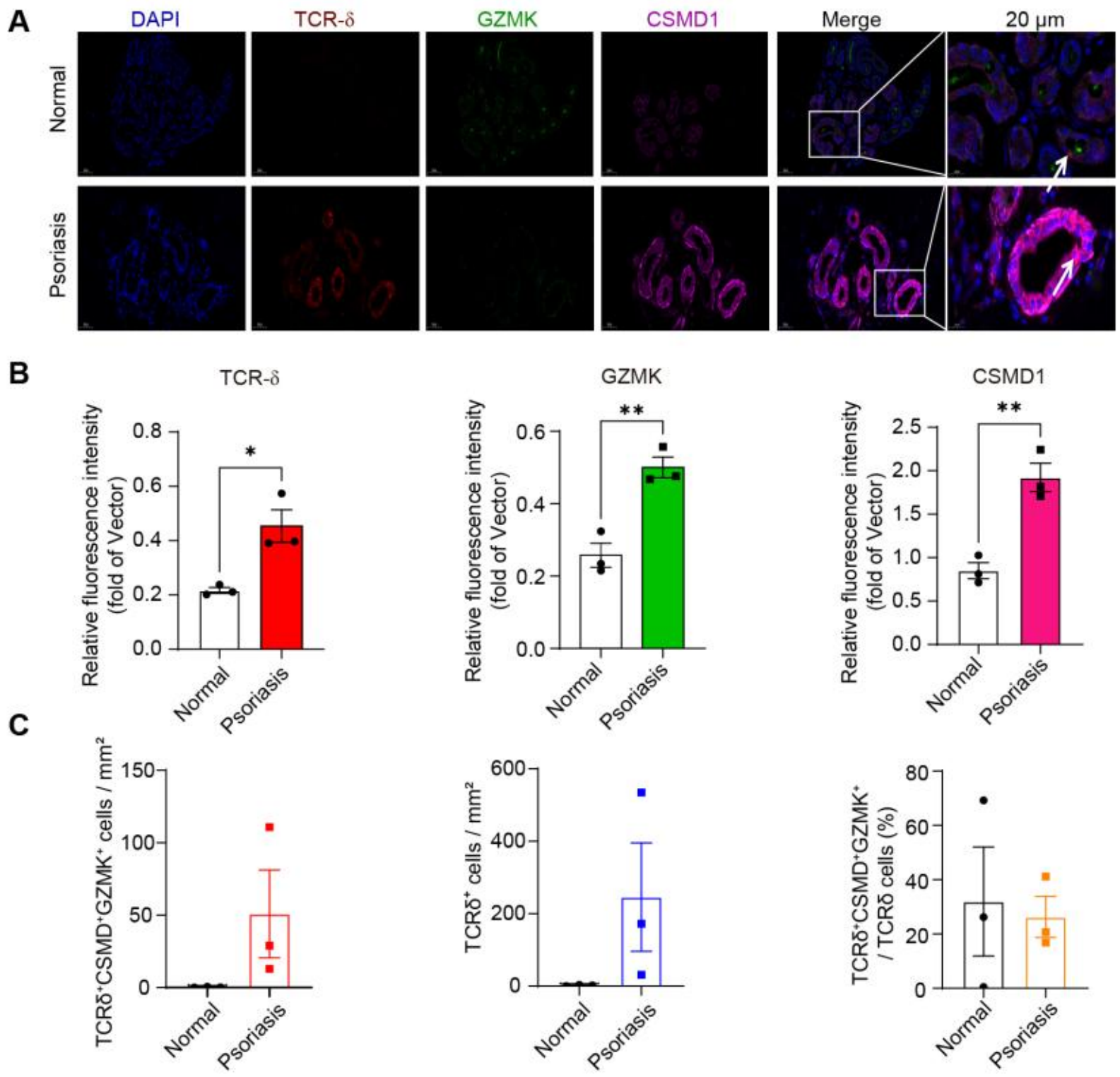

Supplementary Figure 13. Object-level mIF assessment of TCRδ, CSMD1, and GZMK co-detection in normal and psoriatic skin. (A) Representative multiplex immunofluorescence images of Normal skin and psoriatic lesional skin stained for DAPI, TCRδ, GZMK, and CSMD1. Single-channel and merged images are shown. Enlarged regions highlight representative cells with object-level co-detection of TCRδ, GZMK, and CSMD1 signals. White arrows indicate representative $TCR\delta^{+}CSMD1^{+}GZMK^{+}$ cells. Scale bar, 20 μm. (B) Quantification of relative fluorescence intensity for TCRδ, GZMK, and CSMD1 in Normal and Psoriasis skin samples under identical imaging settings. (C) Object-level quantification of $TCR\delta^{+}CSMD1^{+}GZMK^{+}$ cell density, total $TCR\delta^{+}$ cell density, and the percentage of $TCR\delta^{+}CSMD1^{+}GZMK^{+}$ cells among $TCR\delta^{+}$ cells. Cell densities are shown as cells per mm^2. For each donor, the entire tissue section was fully imaged and quantified to generate one donor-level value. Each dot represents one donor. Bars indicate mean ± SEM. Statistical comparisons were performed at the donor level. $*P < 0.05$, $**P < 0.01$.

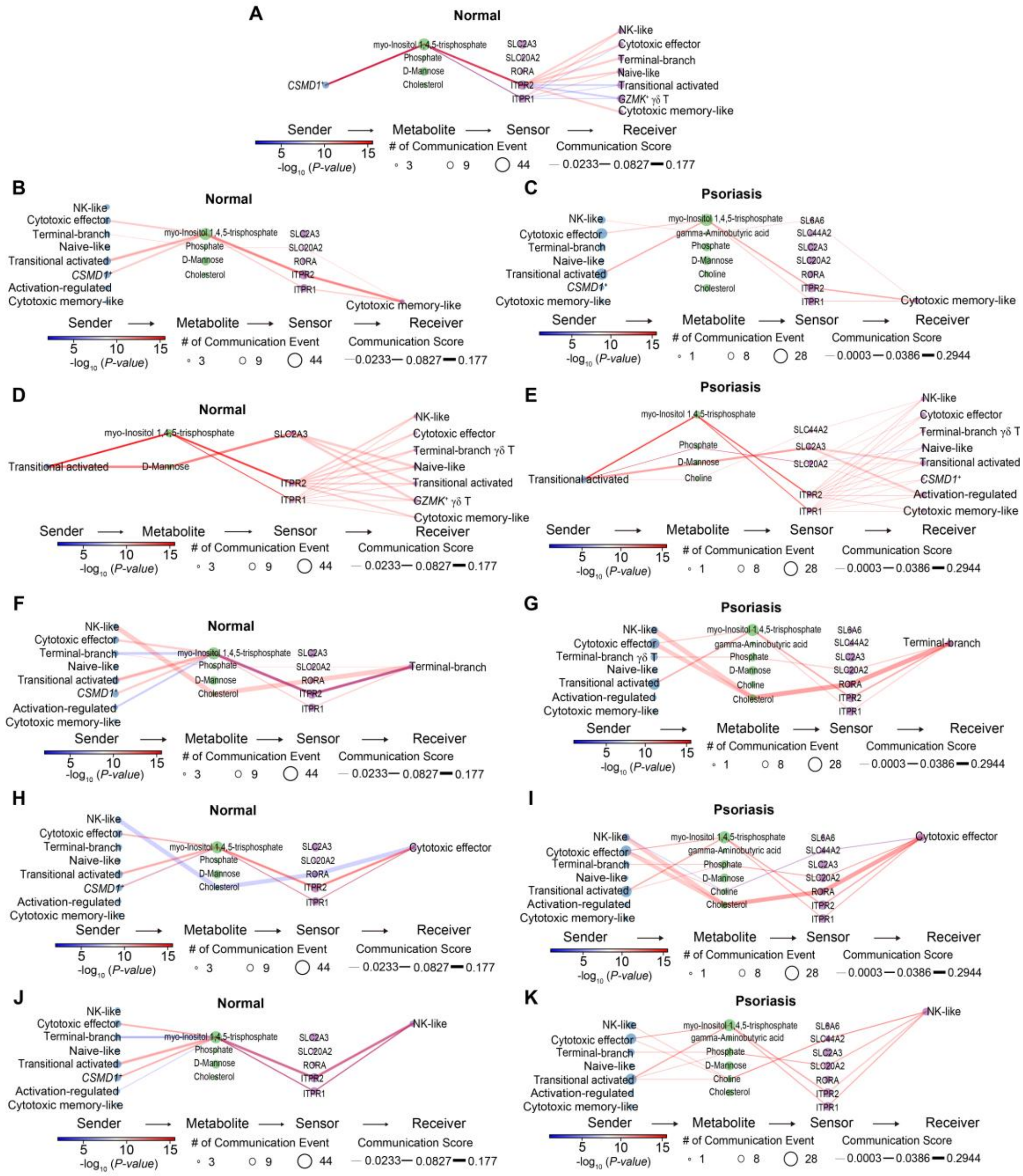

Supplementary Figure 14. Representative mCCC-inferred metabolite–sensor communication routes among γδ T-cell states. (A) Representative network showing *CSMD1*⁺ γδ T cells as senders in normal samples. (B–C) Representative networks showing NK-like γδ T cells as senders in normal (B) and psoriasis (C) samples. (D–E) Representative networks showing Transitional activated γδ T cells as senders in normal (D) and psoriasis (E) samples. (F–G) Representative networks showing metabolite–sensor inputs to Terminal-branch γδ T cells in normal (F) and psoriasis (G) samples. (H–I) Representative networks showing metabolite–sensor inputs to Cytotoxic effector

γδ T cells in normal (H) and psoriasis (I) samples. (J–K) Representative networks showing metabolite–sensor inputs to NK-like γδ T cells in normal (J) and psoriasis (K) samples. Node size indicates the number of communication events, edge width indicates the inferred communication score, and sender color indicates $-\log_{10}$ (P value).

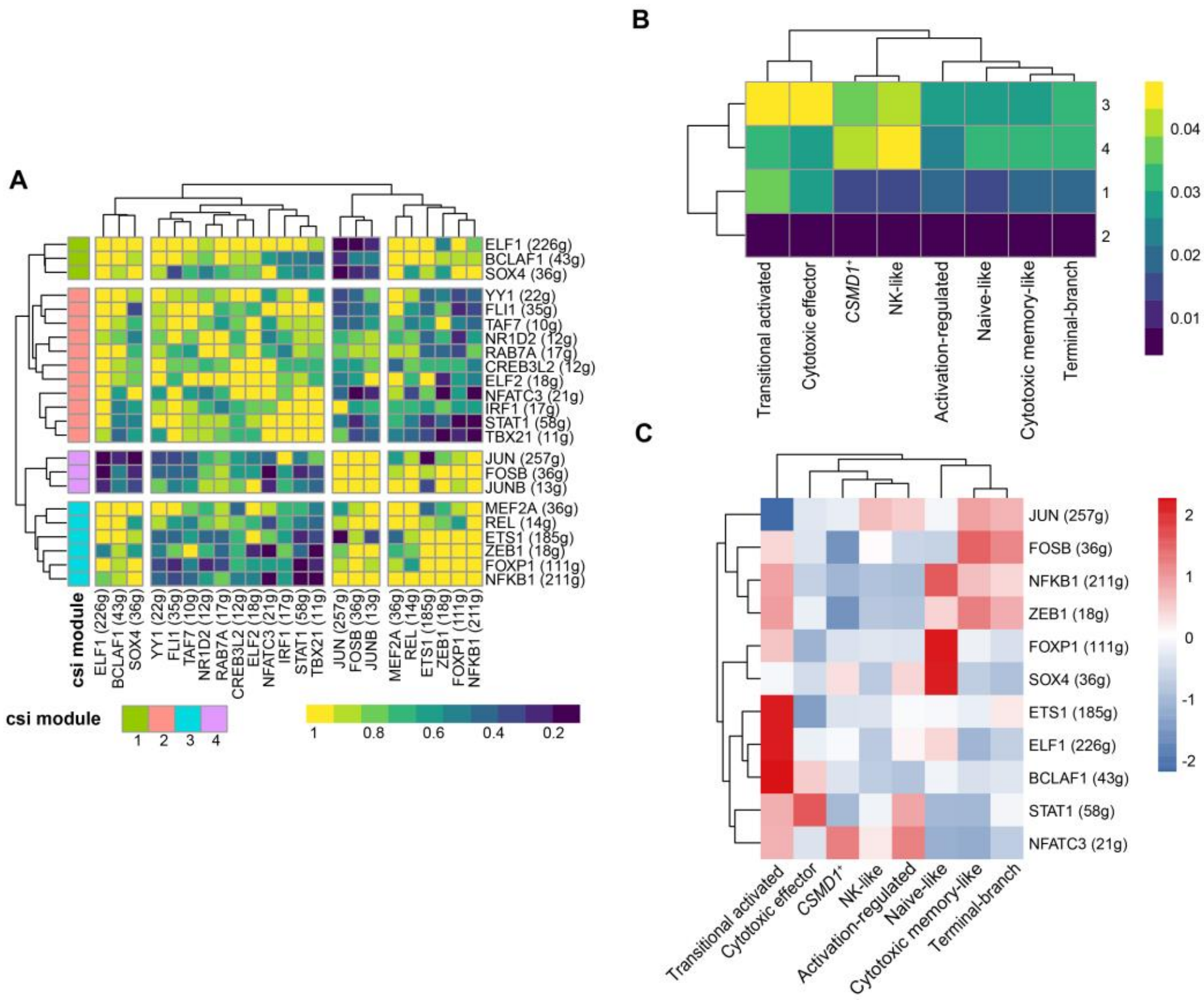

Supplementary Figure 15. Regulon co-activity modules and representative transcription factor feature across circulating γδ T-cell states. (A) Connection specificity index (CSI) heat map showing pairwise relationships among regulons. Based on CSI structure, regulons were grouped into four co-activity modules. (B) Relative distribution of the four CSI modules across γδ T-cell states, showing distinct regulon co-activity patterns associated with different states. (C) Heat map of normalized activity for representative transcription factor regulons across γδ T-cell states, highlighting state-specific regulatory features associated with early, transitional and terminal/cytotoxic programs.

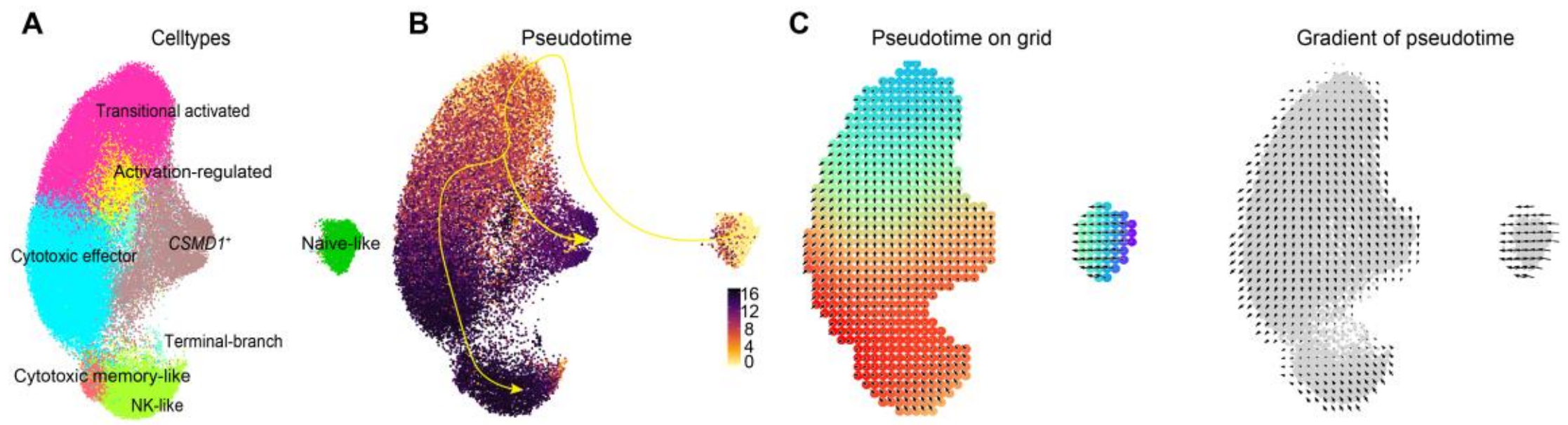

Supplementary Figure 16. Distribution of γδ T-cell states, pseudotime trajectory and construction of the pseudotime gradient field. (A) UMAP representation of γδ T-cell state annotations. Colors indicate the eight annotated γδ T-cell states: Transitional activated, Cytotoxic effector, *CSMD1*$^{+}$, NK-like, Activation-regulated, Naive-like, Cytotoxic memory-like, and Terminal-branch γδ T cells. (B) Pseudotime values overlaid on the same UMAP embedding. Colors indicate progression from early to late pseudotime, and the yellow curve/arrow marks the inferred main trajectory from the early Naive-like state toward late effector-associated states. (C) Mapping of pseudotime values onto a regular grid for construction of a continuous pseudotime field. The left panel shows grid-based pseudotime distribution, and the right panel shows the corresponding pseudotime gradient field. Arrows indicate the direction and relative magnitude of the steepest increase in pseudotime, providing a reference framework for subsequent vector-field and in silico perturbation analyses.

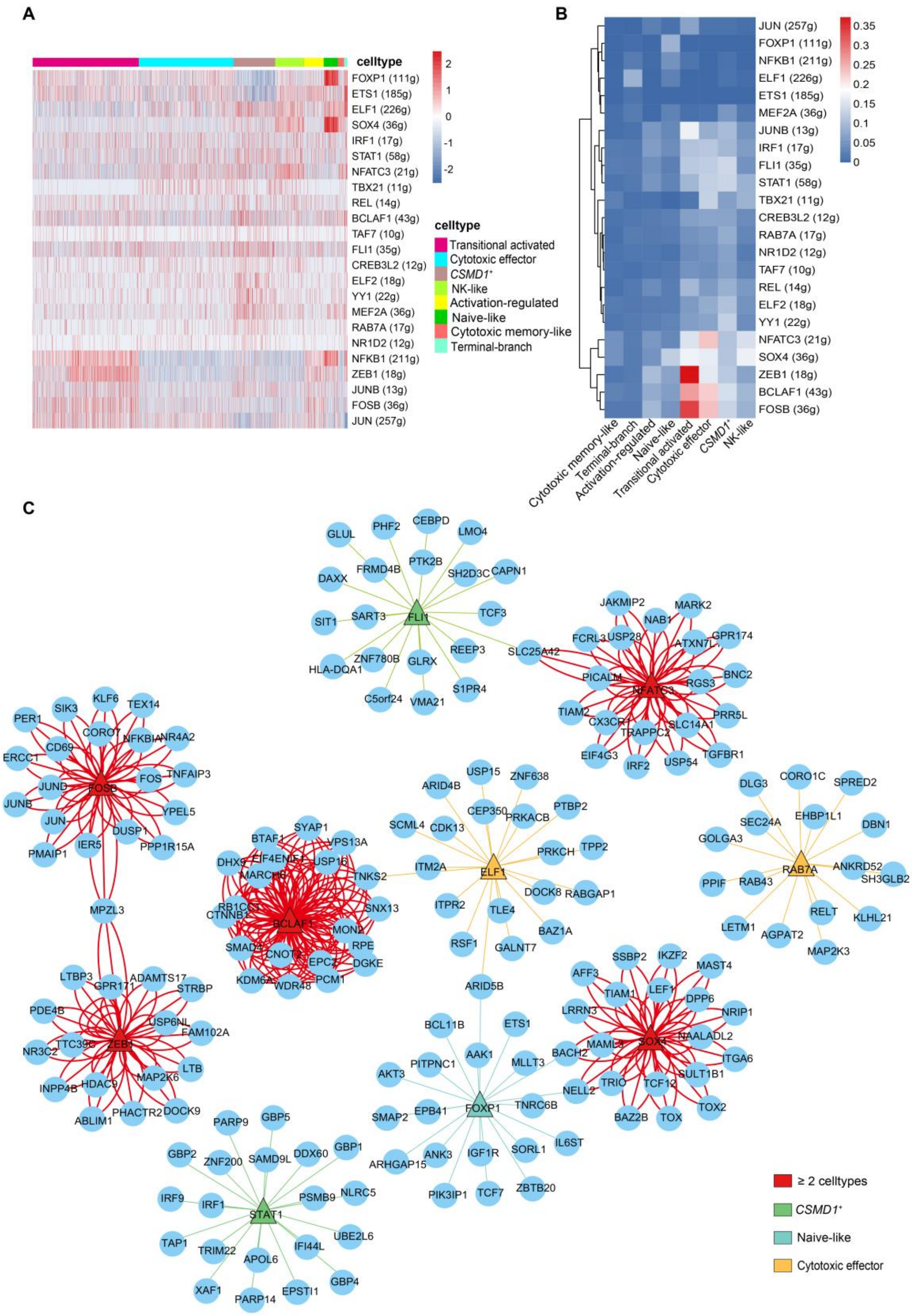

Supplementary Figure 17. Regulon activity landscape and modular regulatory networks across γδ T-cell states. (A) Single-cell heat map showing representative regulon activity inferred by SCENIC/AUCell. Rows indicate representative

transcription factor regulons, with the number of predicted target genes shown in parentheses, and columns represent individual γδ T cells. The top annotation bar indicates γδ T-cell states. Color denotes normalized regulon activity (z score). (B) Summary heat map of regulon activity across annotated γδ T-cell states. Columns represent Cytotoxic memory-like, Terminal-branch, Activation-regulated, Transitional activated, Naive-like, Cytotoxic effector, *CSMD1*$^{+}$, and NK-like γδ T-cell states, and rows represent regulons. Color indicates mean regulon activity. Hierarchical clustering was used to identify transcription factor modules associated with distinct γδ T-cell states. (C) Representative regulon–target gene network showing shared and state-enriched regulatory modules across γδ T-cell states. Central nodes indicate transcription factors and peripheral nodes indicate predicted target genes; edges represent inferred regulatory relationships. Colors denote representative regulon modules enriched in specific γδ T-cell states, including *CSMD1*$^{+}$, Naive-like, and Cytotoxic effector states, whereas red indicates regulon modules shared by two or more γδ T-cell states.
